# Diagnostic accuracy of CBCT versus digital radiography for early detection of peri-implant bone loss: a retrospective study with threshold and observer variability analysis

**DOI:** 10.3389/fdmed.2026.1968914

**Published:** 2026-10-21

**Authors:** Eman Aljoghaiman

**Affiliations:** Preventive Dental Science Department, Periodontics Division, College of Dentistry, Imam Abdulrahman Bin Faisal University, Dammam, Eastern Province, Saudi Arabia

**Keywords:** cone beam computed tomography, dental implants, diagnostic accuracy, digital periapical radiography, peri-implant marginal bone loss, peri-implantitis

## Abstract

**Background:**

Early peri-implant marginal bone loss is clinically important. Digital periapical radiography is routinely used but may have limited sensitivity for subtle osseous changes, whereas CBCT provides three-dimensional visualization.

**Objective:**

To compare CBCT and digital periapical radiography for detecting peri-implant marginal bone loss and evaluate severity-specific sensitivity, observer agreement, and repeatability.

**Methods:**

This retrospective diagnostic accuracy study included 130 implant sites from 100 patients evaluated between January 2018 and December 2025. Three observers independently assessed both modalities. The reference standard classified 73 sites as positive, 55 as negative, and 2 as inconclusive. Binary diagnostic accuracy was based on site-level majority voting (≥2 of 3 observers), while the mean continuous bone-loss measurement across observers was used for primary ROC analysis. Severity-specific sensitivity was assessed for ≤0.5 mm, >0.5–1 mm, and >1 mm bone loss. Interobserver agreement and intraobserver repeatability were evaluated. Patient-clustered generalized estimating equation (GEE) logistic regression accounted for within-patient clustering and repeated observer assessments.

**Results:**

Among 128 evaluable sites, CBCT demonstrated higher sensitivity (97.3% vs. 78.1%) and accuracy (96.9% vs. 87.5%) than digital periapical radiography. Specificity, PPV, and NPV were 96.4%, 97.3%, and 96.4% for CBCT vs. 100.0%, 100.0%, and 77.5%, respectively. AUCs were 0.91 vs. 0.78 (DeLong *p* < 0.001). CBCT sensitivity was 100.0%, 95.0%, and 100.0% across predefined severity categories vs. 46.7%, 80.0%, and 100.0% for digital periapical radiography. Interobserver agreement (*κ* = 0.85 vs. 0.67) and intraobserver repeatability (ICC = 0.93 vs. 0.77) were higher with CBCT. CBCT was associated with higher odds of correct classification than digital periapical radiography (adjusted OR = 1.97, 95% CI: 1.16–3.34; *p* = 0.012).

**Conclusion:**

CBCT showed superior diagnostic performance, particularly for smaller bone-loss lesions. However, radiographic changes of ≤0.5 mm should not be considered definitive evidence of biologically meaningful progression. CBCT should not be used for routine screening of asymptomatic implant sites but considered selectively when clinically justified. Prospective longitudinal studies are warranted.

## Highlights

- CBCT demonstrated higher diagnostic performance than digital periapical radiography for detecting peri-implant marginal bone loss.
- CBCT showed greater sensitivity for smaller bone-loss changes, including the predefined ≤0.5-mm category.
- ROC analysis demonstrated greater discriminatory performance with CBCT than with digital periapical radiography.
- Interobserver agreement and intraobserver repeatability were higher with CBCT than with digital periapical radiography.
- Patient-clustered multi-reader GEE analysis supported the diagnostic advantage of CBCT, supporting its selective use when conventional radiographic findings are equivocal or three-dimensional assessment is clinically justified.

## Introduction

Dental implants are widely regarded as a predictable and effective treatment modality for the rehabilitation of partially and completely edentulous patients, with high long-term survival rates reported in contemporary literature. However, peri-implant diseases remain important biological complications that can adversely affect implant health and longevity. Progressive marginal bone loss is an important feature of peri-implant disease and may be associated with peri-implantitis and, in advanced cases, implant failure. Early diagnosis of peri-implant bone changes is therefore essential for maintaining implant stability and improving long-term clinical outcomes (1, 2).

Radiographic evaluation plays a central role in the assessment and monitoring of peri-implant bone levels. Digital periapical radiography is commonly used for routine implant follow-up because of its low radiation dose, accessibility, rapid acquisition, and cost-effectiveness (3). Nevertheless, as a two-dimensional imaging technique, it represents three-dimensional peri-implant anatomy with inherent limitations related to anatomical superimposition and geometric distortion. In particular, buccal and lingual peri-implant bone defects may remain undetected or may be underestimated until substantial bone destruction has occurred (4, 5). CBCT can overcome some of these limitations by providing multiplanar, three-dimensional visualization of peri-implant bone morphology. Previous experimental and clinical investigations have demonstrated improved detection of peri-implant dehiscence, fenestration, and circumferential bone defects with CBCT compared with conventional radiographic techniques (6, 7). However, CBCT should not be considered a routine replacement for periapical imaging because its use involves greater radiation exposure, potential metal-related artifacts, and higher cost. Accordingly, imaging selection should follow the principles of radiation justification and optimization advocated by ALARA and ALADA (8, 9).

Despite the established advantages of three-dimensional imaging for the visualization of peri-implant defects, an important evidence gap remains concerning the detection of early marginal bone changes. Existing evidence has more consistently addressed larger or clearly defined peri-implant defects, whereas the minimum extent of marginal bone change that can be reliably detected by CBCT compared with digital periapical radiography remains less clearly established. Previous investigations have also included experimental or *in-vitro* models, which may not fully reproduce the anatomical variability and imaging conditions encountered in routine clinical practice (10, 11). Furthermore, the influence of observer experience and the consistency of interpretation across observers warrant further evaluation in clinical datasets. Thus, although CBCT may provide enhanced visualization, whether this translates into improved detection of early marginal bone changes under real-world clinical conditions remains insufficiently defined.

Therefore, the present retrospective diagnostic accuracy study aimed to compare CBCT with digital periapical radiography for the detection of early peri-implant marginal bone loss and to evaluate diagnostic performance across different levels of defect severity. The study additionally assessed observer agreement and reproducibility and examined clinically relevant differences in the detection of subtle and sub-millimeter marginal bone changes. We hypothesized that CBCT would demonstrate higher diagnostic accuracy and greater sensitivity for smaller peri-implant marginal bone changes than digital periapical radiography. The findings were intended to clarify the comparative diagnostic performance of the two imaging modalities and to inform the selective use of CBCT when conventional radiographic findings are equivocal or when three-dimensional assessment is clinically indicated.

## Materials and methods

### Study design

This retrospective diagnostic accuracy study was reported in accordance with the Standards for Reporting Diagnostic Accuracy Studies (STARD 2015) guidelines. The study aimed to compare the diagnostic performance of Cone Beam Computed Tomography (CBCT) and digital periapical radiography for the detection of early peri-implant marginal bone loss.

### Study setting and participants

The study was performed using imaging records retrieved from the institutional Picture Archiving and Communication System (PACS) and radiographic database of a dental teaching hospital. Patients evaluated between January 2018 and December 2025 were screened for eligibility. Potentially eligible participants were identified based on the availability of both CBCT and digital periapical radiographic examinations related to dental implants. A convenience sampling approach was adopted.

The final study population comprised 130 implant sites from 100 unique patients. Of these, 70 patients contributed one implant site and 30 patients contributed two implant sites. Thus, implant sites were nested within patients, and patient-level clustering was accounted for in the inferential analyses. Patient-level and implant-site-level denominators were distinguished throughout the analysis.

### Eligibility criteria

Patients were included if they met the following criteria:

- Presence of at least one dental implant.
- Availability of both CBCT and digital periapical radiographs.
- Imaging examinations performed within an interval of ≤3 months.
- Adequate image quality for diagnostic interpretation.

The exclusion criteria were:

- Motion artifacts or poor-quality images.
- Severe beam-hardening artifacts affecting peri-implant assessment.
- Advanced peri-implant bone loss (>3–4 mm).
- History of recent surgical intervention affecting peri-implant bone levels.
- Incomplete clinical or radiographic records.

The ≤3-month interval was prespecified as the eligibility criterion. The subgroup with an imaging interval of ≤1 month was analyzed separately as an exploratory analysis because only 10 evaluable sites met this criterion.

### Index tests

The index tests evaluated in this study were CBCT and digital periapical radiography. Both index tests were available for all included implant sites and were interpreted independently by the three study observers.

### Reference standard

A reference standard was established for the final diagnostic classification of peri-implant marginal bone loss. Surgical findings were considered the preferred reference standard when surgical verification was available. Of the 130 included implant sites, 45 (34.6%) underwent surgical verification of peri-implant bone status.

For the remaining 85 implant sites (65.4%) without surgical verification, the reference classification was established using a clinical/longitudinal composite reference standard based on probing depth, bleeding on probing, suppuration, and longitudinal marginal bone-level findings. The reference-standard assessment was performed by an experienced oral radiologist who was blinded to the CBCT and digital periapical radiographic interpretations. CBCT interpretation was explicitly excluded from construction of the composite reference standard. The available retrospective records did not permit confirmation of whether digital periapical radiographic findings contributed to the longitudinal component of the composite reference standard; therefore, potential incorporation of DPR-derived information into the longitudinal component cannot be completely excluded.

The final reference-standard classification identified 73 implant sites with peri-implant marginal bone loss, 55 sites without marginal bone loss, and 2 sites with inconclusive reference-standard findings. Because different reference-standard approaches were used according to the availability of surgical verification, differential-verification bias cannot be completely excluded.

A prespecified sensitivity analysis restricted the diagnostic-accuracy analysis to the 45 surgically verified implant sites. One of these sites had an inconclusive reference-standard classification and was therefore excluded from analyses requiring a binary reference outcome, leaving 44 evaluable surgically verified sites.

### CBCT imaging

CBCT examinations were acquired using two CBCT systems under the institutional imaging protocol: Carestream CS 9300 (*n* = 30 examinations) and Planmeca ProMax 3D (*n* = 70 examinations). The Carestream CS 9300 examinations were acquired using a 0.20 mm voxel size, an 8 × 8 cm field of view (FOV), 90 kVp, 4 mA, and an 8 s exposure time, with reconstruction performed using CS 3D Imaging Software. CS MAR (metal-artifact reduction) was enabled when available. The Planmeca ProMax 3D examinations were acquired using a 0.20 mm voxel size, an 8 × 8 cm FOV, 90 kVp, 8 mA, and a 12-second exposure time, with reconstruction performed using Planmeca Romexis and metal-artifact reduction enabled.

Voxel size and FOV were therefore consistent between the two systems, whereas tube current and exposure time differed. These scanner-specific acquisition and reconstruction characteristics were documented as potential sources of imaging-related variability.

Peri-implant bone levels were assessed relative to the implant shoulder, with emphasis on the detection of early crestal bone changes. Cases with severe metal artifacts that prevented reliable assessment were excluded.

### Digital periapical radiography

Digital periapical radiographs were obtained using the paralleling technique with a digital CMOS sensor and standardized institutional exposure protocols. Positioning devices were used to minimize geometric distortion and improve reproducibility. Mesial and distal peri-implant bone levels were assessed relative to the implant platform using digital measurement software.

### Imaging interval

The interval between CBCT and digital periapical radiography was calculated from the recorded acquisition dates of the two examinations. The mean interval between examinations was 1.8 ± 0.7 months. Because the two imaging examinations were not necessarily acquired on the same date, a small amount of true biological change in marginal bone level could theoretically have occurred between examinations and may have contributed to discordant imaging findings. The ≤3-month interval criterion was applied during participant selection to limit this potential temporal effect.

As an exploratory subgroup analysis, implant sites with an imaging interval of ≤1 month were evaluated separately. Only 10 evaluable sites met this criterion; therefore, this subgroup was considered underpowered for definitive inference regarding temporal effects, and its findings were interpreted cautiously.

### Image preparation and observer assessment

All images were anonymized and assigned randomized identification codes prior to evaluation. CBCT scans and digital periapical radiographs were independently assessed by three observers with different levels of clinical experience:

- Expert oral radiologist
- General dentist
- Postgraduate trainee

Before the formal evaluation, all observers underwent a calibration session using 15 representative cases not included in the final study sample to standardize assessment criteria and measurement methodology.

Image interpretation was performed under standardized viewing conditions using calibrated high-resolution monitors in a dimly lit environment and dedicated imaging software.

Observers were blinded to:

- Clinical findings
- Reference-standard results
- Results of the alternative imaging modality
- Assessments made by other observers

Each observer independently recorded:

- Presence or absence of peri-implant marginal bone loss
- Confidence score using a 5-point Likert scale
- Estimated extent of marginal bone loss in millimeters

For the primary site-level binary diagnostic-accuracy analysis, the final classification for each imaging modality was determined by majority vote among the three observers. A site was classified as positive when at least two of the three observers recorded a positive interpretation and as negative when at least two recorded a negative interpretation. Accordingly, the single CBCT and digital periapical radiography classifications presented in the site-level diagnostic-accuracy tables represent the corresponding majority-vote classifications rather than the assessment of a single designated observer or an average of the continuous measurements.

Observer-specific diagnostic performance was additionally calculated from the original binary interpretations of each observer and is reported separately to characterize reader-level variability. Observer experience was not used as an inferential predictor because each experience category was represented by a single observer and was therefore completely confounded with observer identity. The three observer assessments of the same implant site were not treated as independent observations in the inferential analysis.

The estimated marginal bone-loss measurement was recorded as a continuous variable. For each implant site and imaging modality, the mean estimated marginal bone-loss value across the three observers was calculated and used as the continuous predictor in the site-level receiver operating characteristic (ROC) analysis. This averaging procedure was used only for the continuous ROC analysis and was not used to derive the primary binary diagnostic classifications.

To account for the hierarchical structure of the data, including multiple implant sites contributed by some patients and repeated assessments across observers and imaging modalities, the inferential multi-reader analysis used a patient-clustered generalized estimating equation (GEE) logistic model with robust standard errors. Observer identity was included as an adjustment factor, whereas observer experience was excluded because of its complete confounding with observer identity.

To evaluate intraobserver reliability, repeat assessments were performed after a two-week interval using randomized image sequences.

Interobserver agreement for the ordinal marginal bone-loss categories was assessed using weighted Cohen's kappa for pairwise observer comparisons. Intraobserver reliability of the continuous estimated marginal bone-loss measurements was assessed using a two-way random-effects, single-measure intraclass correlation coefficient for absolute agreement (ICC[2,1]. Ninety-five percent confidence intervals (CIs) were calculated for the agreement estimates.

### Bone loss measurement and categorization

For each implant site, marginal bone level was assessed at the baseline examination and the subsequent current examination, using the implant shoulder/platform as the anatomical reference. The baseline measurement represented the earliest recorded marginal bone-level measurement available for longitudinal comparison, whereas the current measurement represented the corresponding marginal bone-level measurement at the subsequent examination. Marginal bone loss was calculated as the change in marginal bone level between the two examinations:

Marginalboneloss(mm)=currentmarginalbonelevel−baselinemarginalbonelevel.

The calculated change in marginal bone level was categorized as follows:

- **Category 0:** No bone loss
- **Category 1:** ≤0.5 mm
- **Category 2:** >0.5–1 mm
- **Category 3:** >1 mm

These predefined severity categories were used to describe the distribution of marginal bone loss and to evaluate diagnostic performance across clinically relevant severity levels. They were distinct from the data-driven diagnostic threshold derived from ROC analysis using the Youden index. For the primary binary diagnostic-accuracy analysis, the independently recorded binary observer interpretations for each imaging modality were compared with the reference-standard classification. The continuous marginal bone-loss measurements estimated independently by the observers were analyzed separately, with the mean value across observers used as the site-level predictor for the primary ROC analysis. The reference-standard severity categories were based on the calculated longitudinal change in marginal bone level.

### Statistical analysis

Statistical analyses were performed using R statistical software version 4.5.2 (R Foundation for Statistical Computing, Vienna, Austria) and SAS software version 9.4 (SAS Institute Inc., Cary, NC, USA). The implant site was the primary unit of diagnostic analysis, whereas patient identification was retained to account for the non-independence of multiple implant sites contributed by the same patient. Patient-level and implant-site-level denominators were distinguished throughout the analyses.

### Diagnostic accuracy and ROC analysis

For the primary diagnostic accuracy analysis, the binary reference-standard classification was compared with the site-level binary classification for each imaging modality using the majority-vote approach described above. Sensitivity, specificity, positive predictive value (PPV), negative predictive value (NPV), diagnostic accuracy, and diagnostic odds ratio (DOR) were calculated from 2 × 2 contingency tables with corresponding 95% confidence intervals. Observer-specific diagnostic accuracy was additionally calculated from the original binary interpretations of each observer to characterize reader-level variability. These individual observer assessments were not treated as independent patient-level observations.

Continuous observer-estimated marginal bone-loss measurements were analyzed separately from the binary diagnostic classifications. ROC curves were generated using the continuous estimated marginal bone-loss measurement, rather than the 5-point confidence score or categorical severity classification. For each implant site and imaging modality, the mean estimated marginal bone-loss value across the three observers was calculated and used as the continuous predictor for the site-level ROC analysis. This averaging was restricted to the ROC analysis and was not used to derive the primary binary diagnostic classification.

Because both imaging modalities were evaluated at the same implant sites and some patients contributed more than one implant site, the paired modality comparison was performed using a patient-clustered generalized estimating equation (GEE) logistic model. Diagnostic correctness was modeled as the binary outcome, with imaging modality as the principal predictor and patient specified as the clustering variable. For the multi-reader analysis, observer identity, implant location, and reference-standard bone-loss severity were included as adjustment factors. Observer experience was not included as an inferential predictor because each experience category was represented by only one observer and was therefore completely confounded with observer identity. Robust standard errors accounted for within-patient correlation. The direction and number of discordant site-level majority-vote classifications were also reported descriptively.

The revised GEE approach was used instead of a random-effects model for observer because only three observers were available, making estimation of an observer-level random-effect variance potentially unstable. The GEE model therefore accounted for patient-level clustering through robust sandwich standard errors while treating observer identity as an adjustment factor rather than estimating an observer random effect.

Reader-specific ROC analyses were performed separately for each observer using the continuous estimated marginal bone-loss measurement as the diagnostic predictor and the binary reference-standard classification as the outcome. AUCs were calculated separately for CBCT and digital periapical radiography, and 95% confidence intervals were estimated using nonparametric bootstrap resampling.

For the primary site-level ROC analysis, receiver operating characteristic (ROC) curves were generated using the mean continuous estimated marginal bone-loss measurement across the three observers as the site-level predictor. AUCs and 95% CIs were calculated using the DeLong method, and the difference between the correlated AUCs for CBCT and digital periapical radiography was assessed using the DeLong test.

The predefined bone-loss categories (no bone loss, ≤0.5 mm, >0.5–1 mm, and >1 mm) were used for descriptive and severity-specific analyses and were not used as ROC thresholds. The ROC-derived optimal diagnostic threshold was determined independently from the continuous bone-loss measurement using the Youden index. Thus, the predefined ≤0.5 mm category represented a clinically defined severity group, whereas the Youden-derived threshold represented a data-driven diagnostic cutoff for the binary reference-standard outcome.

### Comparison of diagnostic performance

Because the same implant sites were evaluated using both imaging modalities, the paired nature of the site-level classifications was preserved in the analysis. The numbers and direction of discordant site-level majority-vote classifications were reported descriptively to characterize the paired differences between CBCT and digital periapical radiography. The inferential comparison of diagnostic correctness was performed using the patient-clustered GEE model described below.

### Multi-reader and multi-case analysis

Each of the three observers independently evaluated the same implant sites using both imaging modalities. Observer-specific diagnostic accuracy measures were calculated from the individual observer assessments, whereas the primary site-level ROC analysis used the mean continuous estimated marginal bone-loss measurement across the three observers.

To account for the hierarchical structure of the multi-reader, multi-case data while avoiding confounding between observer experience and observer identity, a generalized estimating equation (GEE) logistic model was used with diagnostic correctness as the binary outcome. Imaging modality, observer identity, implant location, and reference-standard bone-loss severity were included as adjustment factors, and patient was specified as the clustering variable. Robust sandwich standard errors were used to account for within-patient correlation arising from multiple implant sites contributed by the same patient and repeated modality assessments. Observer experience was not included as an inferential predictor because each experience category was represented by only one observer and was therefore completely confounded with observer identity.

As a model-specification check, the adjusted patient-clustered GEE model was compared with a simpler patient-clustered GEE model containing imaging modality as the sole predictor. The direction and magnitude of the modality effect were essentially unchanged between the simpler and adjusted specifications.

Observer-specific differences were presented descriptively and were not interpreted as independent effects of professional experience.

The regression analysis included 128 implant sites with definitive reference-standard classifications. The two sites with inconclusive reference-standard classifications were excluded from analyses requiring a binary reference outcome. These 128 sites were contributed by 100 unique patients, of whom 70 contributed one implant site and 30 contributed two implant sites.

Model convergence was assessed before interpretation of the parameter estimates. Because the revised analysis did not include an observer random effect, no observer-level random-effect variance was estimated. Confidence intervals for the GEE estimates were derived from the robust sandwich covariance matrix clustered at the patient level. Thus, variance estimation accounted for the 100 independent patient clusters rather than treating the 768 observer-level records as independent observations.

### Observer agreement and Reliability

Interobserver agreement for the ordinal bone-loss categories was assessed using weighted Cohen's kappa for pairwise comparisons among the three observers, separately for each imaging modality. Agreement estimates and their 95% confidence intervals were reported for each observer pair.

Intraobserver repeatability of the continuous estimated marginal bone-loss measurements was assessed using a two-way random-effects, single-measure intraclass correlation coefficient for absolute agreement [ICC(2,1)], based on the first and repeat assessments obtained after the two-week interval. The ICC and corresponding 95% confidence interval were reported separately for each imaging modality and, where applicable, for individual observers.

### Subgroup and sensitivity analyses

Subgroup analyses were performed according to implant location and predefined early marginal bone-loss severity (≤1 mm). Observer-specific diagnostic performance was reported descriptively; observer experience level was not evaluated as an independent inferential subgroup because each experience category was represented by only one observer and was therefore completely confounded with observer identity.

A prespecified sensitivity analysis was performed by restricting the diagnostic accuracy analysis to the 45 implant sites with surgical verification of peri-implant bone status. This analysis was undertaken to assess the robustness of the diagnostic accuracy estimates with respect to reference-standard composition and to provide an assessment less dependent on the clinical/longitudinal composite reference standard.

An exploratory sensitivity analysis was additionally performed by restricting the diagnostic accuracy analysis to implant sites with a shorter interval (≤1 month) between CBCT and digital periapical examinations, to evaluate the potential influence of the time interval between the index tests. Because only 10 evaluable implant sites met this criterion, this subgroup analysis was considered underpowered for definitive inference regarding temporal effects and was interpreted cautiously.

### Missing and indeterminate data

All three observers provided definitive binary interpretations (positive or negative) for both CBCT and digital periapical radiography at all 128 implant sites with a definitive reference-standard classification. No inconclusive index-test interpretations were recorded for any observer or imaging modality. The number of inconclusive index-test interpretations was 0 for Observer 1, 0 for Observer 2, and 0 for Observer 3 for CBCT, and 0 for Observer 1, 0 for Observer 2, and 0 for Observer 3 for digital periapical radiography. Consequently, no site-level index-test finding required exclusion, reclassification, or conversion to a binary outcome, and all 128 evaluable sites had at least two concordant definitive observer interpretations for the prespecified majority-vote classification. Because no inconclusive index-test interpretations were recorded, there were no corresponding voting patterns requiring alternative classification.

As sensitivity analyses, sites with any indeterminate index-test interpretation were excluded, and indeterminate index-test interpretations were separately treated as incorrect classifications. Because no indeterminate index-test interpretations were present, both sensitivity analyses retained all 128 evaluable sites and produced results identical to the primary analysis.

The two sites with inconclusive reference-standard classifications were excluded from the 2 × 2 contingency tables and other analyses requiring a definitive binary reference outcome, leaving 128 evaluable implant sites. Missing observations were handled using complete-case analysis for the relevant analysis.

### Statistical significance

A two-sided *P* value <0.05 was considered statistically significant. Confidence intervals were two-sided 95% intervals unless otherwise specified.

### Sample size consideration

The required sample size was determined prospectively for a paired diagnostic-accuracy comparison between CBCT and digital periapical radiography. The calculation assumed an anticipated AUC of 0.88 for CBCT and 0.75 for digital periapical radiography, reflecting the anticipated difference in diagnostic discrimination informed by previous comparative evidence demonstrating superior detection and measurement of peri-implant bone defects with CBCT compared with intraoral radiography (6). A two-sided *α* of 0.05% and 80% power were specified. A reference-standard-positive prevalence of 57.0% and a paired correlation of 0.75 between the two imaging classifications were specified as planning assumptions. The calculation was performed using R statistical software (version 4.5.2) with a normal-approximation approach for the difference between two correlated AUCs. Variance components were based on the Hanley–McNeil formulation (12), with covariance estimated from the assumed paired correlation. The estimated minimum requirement was 49 evaluable implant sites. Allowing approximately 5% for potential exclusions or incomplete assessments, the target sample size was 52 implant sites. The final dataset comprised 130 implant sites from 100 unique patients, substantially exceeding the prespecified minimum.

The prospective sample-size calculation was based on the number of evaluable implant sites and did not include an explicit design-effect adjustment for clustering of implant sites within patients or for the multi-reader structure. Because the final study substantially exceeded the prespecified minimum, clustering and repeated assessments were addressed analytically using patient-clustered methods rather than by retrospectively modifying the original sample-size calculation.

### Ethical considerations

The study protocol was approved by the Imam Abdulrahman Bin Faisal University Institutional Review Board under approval number IRB-2026-02-0541 prior to data collection. Owing to the retrospective nature of the study and the use of anonymized patient data, the requirement for informed consent was waived by the Institutional Review Board. All procedures were conducted in accordance with the ethical principles of the Declaration of Helsinki.

## Results

### Participant flow and baseline characteristics

A total of 168 implant sites were initially screened from the institutional radiographic database between January 2018 and December 2025 (Figure 1). Of these, 38 implant sites were excluded because of poor image quality or artifacts (*n* = 14), advanced peri-implant bone loss >3–4 mm (*n* = 11), incomplete clinical records (*n* = 8), or an imaging interval exceeding 3 months (*n* = 5). Consequently, 130 implant sites from 100 unique patients fulfilled the eligibility criteria and underwent both CBCT and digital periapical radiographic evaluation. Of the 100 unique patients, 70 contributed one implant site and 30 contributed two implant sites.

**Figure 1:**
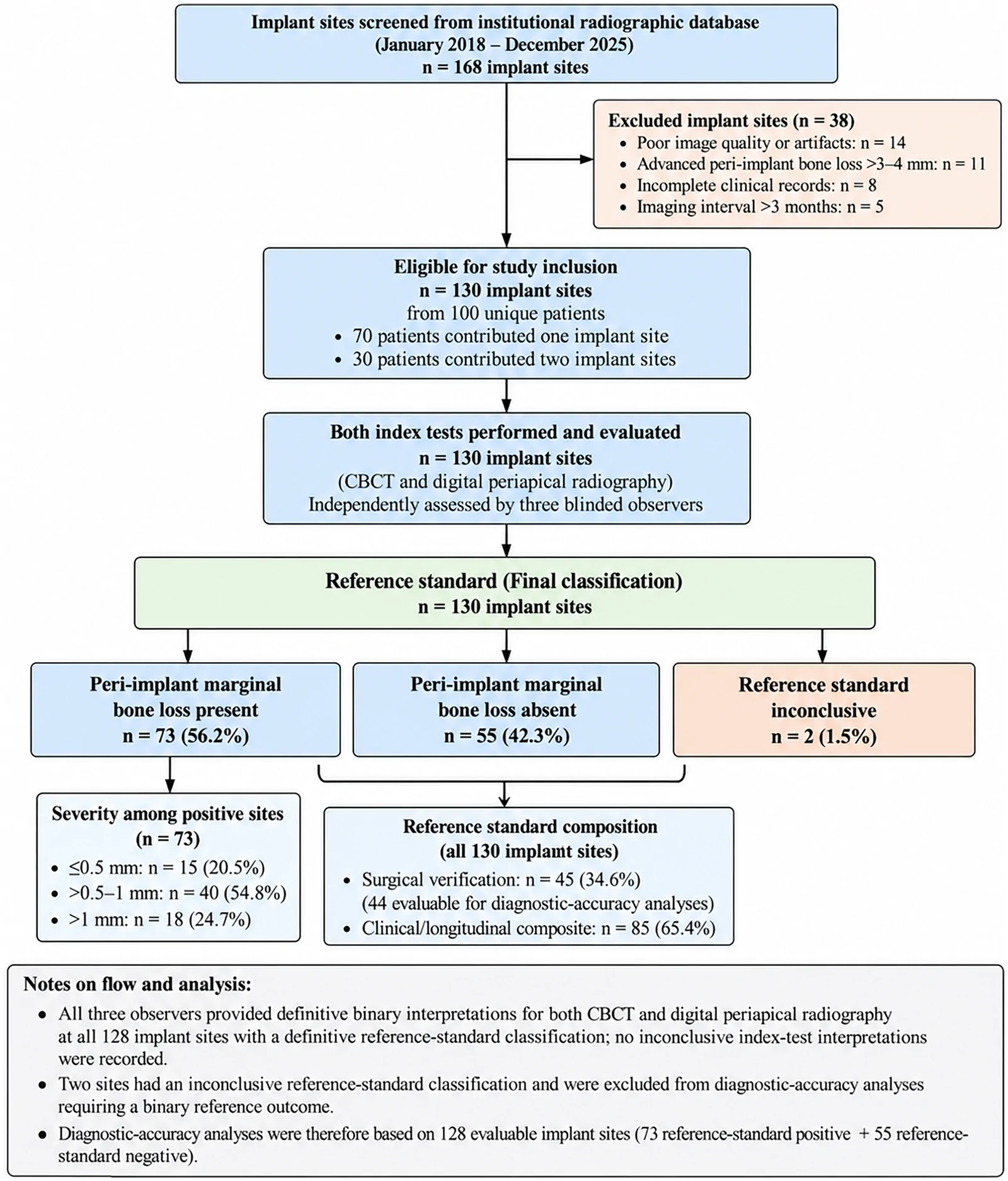
STARD flow diagram illustrating participant screening, eligibility assessment, exclusion criteria, index testing, reference standard evaluation, and final diagnostic classification of implant sites included in the study.

Among the 130 included implant sites, the interval between CBCT and digital periapical radiography was 1.8 ± 0.7 months (median, 1.73 months; range, 0.30–2.99 months). In the exploratory subgroup with an imaging interval of ≤1 month, 10 evaluable implant sites were available. Both CBCT and digital periapical radiography correctly classified all 10 sites (sensitivity, specificity, and accuracy, 100.0% for each modality); however, this subgroup was considered underpowered for definitive inference regarding temporal effects.

All 130 included implant sites underwent both index tests and were independently assessed by three blinded observers. The reference standard classified 73 implant sites as positive for peri-implant marginal bone loss, 55 as negative, and 2 as inconclusive. The two sites with inconclusive reference-standard classifications were retained in the overall study cohort but were excluded from the primary binary diagnostic-accuracy analyses. The reference-standard classification was determined independently of the site-level majority-vote classification of the index tests; potential incorporation of DPR-derived information into the longitudinal component of the composite reference standard could not, however, be completely excluded.

The mean age was 50.9 ± 13.3 years. Patient-level and implant-site-level denominators were distinguished throughout the analysis; demographic variables were summarized at the patient level, whereas implant location and imaging outcomes were summarized at the implant-site level. Implant distribution included maxillary anterior sites in 30 cases (23.1%), maxillary posterior sites in 38 cases (29.2%), mandibular anterior sites in 32 cases (24.6%), and mandibular posterior sites in 30 cases (23.1%) (Table 1).

**Table 1:** Baseline demographic and implant-site characteristics.

Variable	Value
Unique patients, *n*	100
Implant sites included, *n*	130
Patients contributing one implant site, *n* (%)	70 (70.0)
Patients contributing two implant sites, *n* (%)	30 (30.0)
Mean age, years, mean ± SD	50.9 ± 13.3
Sex distribution	
Male, *n* (%)	60 (60.0)
Female, *n* (%)	40 (40.0)
Implant location, *n* (%)	
Maxillary anterior	30 (23.1)
Maxillary posterior	38 (29.2)
Mandibular anterior	32 (24.6)
Mandibular posterior	30 (23.1)
Interval between imaging examinations, months, median (range)	1.73 (0.30–2.99)
Reference-standard classification, *n* (%)	
Peri-implant marginal bone loss present	73 (56.2)
Peri-implant marginal bone loss absent	55 (42.3)
Reference standard inconclusive	2 (1.5)
Severity among reference-standard-positive sites, *n* (%)	
≤0.5 mm	15 (20.5)
>0.5–1 mm	40 (54.8)
>1 mm	18 (24.7)

Percentages for sex are calculated using the 100 unique patients. Percentages for implant location and the overall reference-standard classification are calculated using the 130 implant sites. Percentages for bone-loss severity are calculated among the 73 reference-standard-positive implant sites. The two inconclusive reference-standard sites were not assigned to a bone-loss severity category. Seventy patients contributed one implant site, whereas 30 patients contributed two implant sites.

Among the 73 reference-standard-positive implant sites, 15 sites (20.5%) had ≤0.5 mm bone loss, 40 sites (54.8%) had >0.5–1 mm bone loss, and 18 sites (24.7%) had >1 mm bone loss (Table 1). These categories accounted for all 73 reference-standard-positive sites.

### Diagnostic accuracy

CBCT demonstrated higher diagnostic performance than digital periapical radiography for the detection of peri-implant marginal bone loss. Cross-tabulation based on the site-level majority-vote classifications showed that CBCT correctly identified 71 of 73 reference-standard-positive implant sites, with 2 false-negative and 2 false-positive findings, whereas digital periapical radiography correctly identified 57 of 73 reference-standard-positive sites, with 16 false-negative and no false-positive findings (Tables 2, 3).

**Table 2:** Cross-tabulation of site-level majority-vote CBCT findings against the reference standard.

CBCT Majority-vote result	Bone loss present	Bone loss absent	Reference-standard inconclusive	Total
Positive	71	2	0	73
Negative	2	53	0	55
Inconclusive	0	0	2	2
Total	73	55	2	130

Site-level CBCT classification was determined by majority vote among the three observers (≥2 of 3 observers). The two sites with an inconclusive reference-standard classification were excluded from the primary diagnostic accuracy analysis requiring a binary reference outcome. Among the remaining 128 evaluable implant sites, 73 were reference-standard positive and 55 were reference-standard negative. All three observers provided definitive binary CBCT interpretations for the 128 evaluable sites; therefore, no unresolved site-level indeterminate CBCT classifications remained after application of the majority-vote rule.

**Table 3:** Cross-tabulation of site-level majority-vote digital periapical radiography findings against the reference standard.

Digital periapical radiography majority-vote result	Bone loss present	Bone loss absent	Reference-standard inconclusive	Total
Positive	57	0	0	57
Negative	16	55	0	71
Inconclusive	0	0	2	2
Total	73	55	2	130

Site-level digital periapical radiography classification was determined by majority vote among the three observers (≥2 of 3 observers). The two sites with an inconclusive reference-standard classification were excluded from the primary diagnostic accuracy analysis requiring a binary reference outcome. Among the remaining 128 evaluable implant sites, 73 were reference-standard positive and 55 were reference-standard negative. All three observers provided definitive binary digital periapical radiography interpretations for the 128 evaluable sites; therefore, no unresolved site-level indeterminate digital periapical radiography classifications remained after application of the majority-vote rule.

Among the 128 evaluable implant sites, CBCT demonstrated a sensitivity of 97.3% (71/73; 95% CI: 90.5%–99.2%), specificity of 96.4% (53/55; 95% CI: 87.7%–99.0%), positive predictive value of 97.3% (71/73; 95% CI: 90.5%–99.2%), and negative predictive value of 96.4% (53/55; 95% CI: 87.7%–99.0%). Digital periapical radiography demonstrated a sensitivity of 78.1% (57/73; 95% CI: 67.3%–86.0%), specificity of 100.0% (55/55; 95% CI: 93.5%–100.0%), positive predictive value of 100.0% (57/57; 95% CI: 93.7%–100.0%), and negative predictive value of 77.5% (55/71; 95% CI: 66.5%–85.6%). Overall diagnostic accuracy was 96.9% (124/128; 95% CI: 92.2%–98.8%) for CBCT and 87.5% (112/128; 95% CI: 80.7%–92.2%) for digital periapical radiography (Table 4).

**Table 4:** Diagnostic performance of CBCT and digital periapical radiography.

Parameter	CBCT	Digital periapical radiography
Sensitivity, % (95% CI)	97.3 (90.5–99.2)	78.1 (67.3–86.0)
Specificity, % (95% CI)	96.4 (87.7–99.0)	100.0 (93.5–100.0)
Positive Predictive Value, % (95% CI)	97.3 (90.5–99.2)	100.0 (93.7–100.0)
Negative Predictive Value, % (95% CI)	96.4 (87.7–99.0)	77.5 (66.5–85.6)
Diagnostic Accuracy, % (95% CI)	96.9 (92.2–98.8)	87.5 (80.7–92.2)
Diagnostic Odds Ratio	940.75	Not estimable
AUC (95% CI)	0.91 (0.84–0.97)	0.78 (0.68–0.89)
DeLong test (CBCT vs. digital periapical radiography)	*p* < 0.001	—

Diagnostic performance estimates were calculated among 128 evaluable implant sites, after exclusion of two sites with inconclusive reference-standard classifications. Binary classifications for both imaging modalities were based on site-level majority vote among the three observers (positive when ≥2 of 3 observers classified the site as positive and negative when ≥2 classified the site as negative). For CBCT, the 2 × 2 contingency table comprised TP = 71, FN = 2, TN = 53, and FP = 2. For digital periapical radiography, the corresponding values were TP = 57, FN = 16, TN = 55, and FP = 0. Sensitivity, specificity, positive predictive value (PPV), negative predictive value (NPV), and diagnostic accuracy are presented with 95% confidence intervals (CIs). Confidence intervals for these proportions were calculated using the Wilson method. The diagnostic odds ratio (DOR) for digital periapical radiography was not estimable because no false-positive findings were observed. AUC values and their 95% confidence intervals were calculated using the DeLong method. The difference between the correlated AUCs for CBCT and digital periapical radiography was also assessed using the DeLong method.

Diagnostic odds ratio analysis further demonstrated the high discriminatory performance of CBCT (DOR = 940.75; 95% CI: 128.34–6,896.08). A finite diagnostic odds ratio could not be estimated for digital periapical radiography because no false-positive findings were observed (FP = 0); consequently, its diagnostic odds ratio was mathematically infinite and was therefore reported as not estimable in Table 4.

Because the same implant sites were evaluated using both imaging modalities, the paired nature of the site-level classifications was preserved in the analysis. There were 18 discordant sites, comprising 17 sites classified as positive by CBCT but negative by digital periapical radiography and 1 site classified as negative by CBCT but positive by digital periapical radiography. The inferential comparison of diagnostic correctness was performed using a patient-clustered generalized estimating equation (GEE) logistic model, with diagnostic correctness as the binary outcome, imaging modality as the principal predictor, and patient as the clustering variable. CBCT was associated with significantly higher odds of correct diagnostic classification than digital periapical radiography (OR = 4.43, 95% CI: 1.48–13.20; *p* = 0.008).

### Receiver operating characteristic curve analysis

Receiver operating characteristic (ROC) curve analysis demonstrated higher discriminatory performance of CBCT than digital periapical radiography for detecting peri-implant marginal bone loss (Figure 2). The ROC curves were generated using the continuous estimated marginal bone-loss measurements, rather than the 5-point confidence scores or the predefined categorical severity classifications. For each implant site and imaging modality, the mean estimated marginal bone-loss value across the three observers was used as the continuous predictor for the primary site-level ROC analysis.

**Figure 2:**
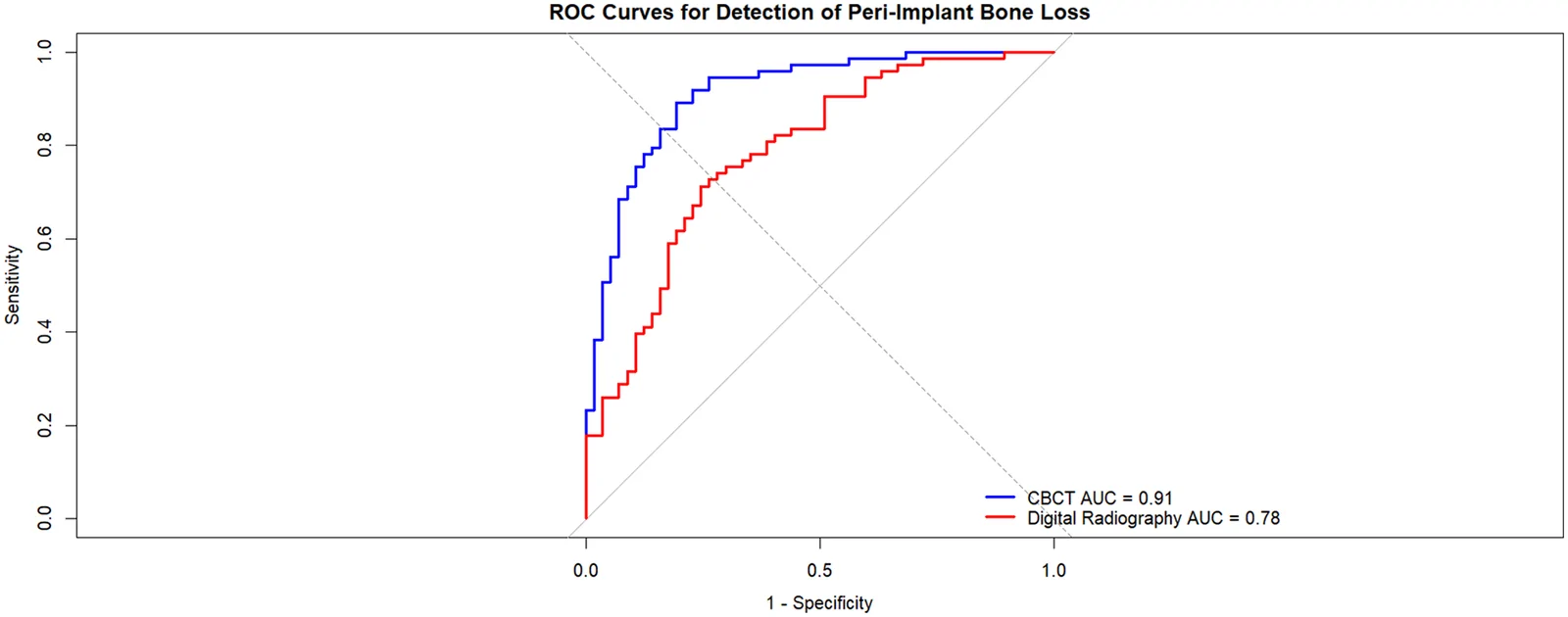
Receiver operating characteristic (ROC) curves for CBCT and digital periapical radiography. ROC curves were generated using the continuous estimated marginal bone-loss measurements. For each implant site and imaging modality, the mean estimated marginal bone-loss measurement across the three observers was used as the continuous site-level predictor. The area under the ROC curve (AUC) was 0.91 (95% CI: 0.84–0.97) for CBCT and 0.78 (95% CI: 0.68–0.89) for digital periapical radiography. The difference between the AUCs was statistically significant (DeLong test, *p* < 0.001). The ROC analysis was separate from the primary binary diagnostic-accuracy analysis, in which classifications were based on site-level majority vote among the three observers.

The area under the ROC curve (AUC) for CBCT was 0.91 (95% CI: 0.84–0.97), whereas digital periapical radiography demonstrated an AUC of 0.78 (95% CI: 0.68–0.89) (Table 4). The difference between the correlated AUCs was statistically significant according to the DeLong test (*p* < 0.001) (Figure 2). These findings indicate that CBCT demonstrated greater ability to discriminate between implant sites classified by the reference standard as having or not having peri-implant marginal bone loss.

Reader-specific ROC analyses were performed separately for each observer using that observer's continuous estimated marginal bone-loss measurement as the diagnostic predictor. The expert oral radiologist demonstrated an AUC of 0.91 (95% bootstrap CI: 0.86–0.95) for CBCT and 0.75 (95% bootstrap CI: 0.66–0.83) for digital periapical radiography. For the general dentist, the corresponding AUCs were 0.89 (95% bootstrap CI: 0.84–0.94) and 0.77 (95% bootstrap CI: 0.69–0.85), respectively. For the postgraduate trainee, the AUCs were 0.92 (95% bootstrap CI: 0.87–0.96) for CBCT and 0.77 (95% bootstrap CI: 0.68–0.84) for digital periapical radiography. Thus, higher reader-specific discriminatory performance was observed consistently for CBCT across all three observers. These reader-specific analyses were descriptive and were not interpreted as independent effects of professional experience.

The predefined bone-loss categories (≤0.5 mm, >0.5–1 mm, and >1 mm) were used separately for severity-specific analyses and were not derived from the ROC analysis. A data-driven diagnostic cut-off was determined separately from the continuous ROC predictor using the Youden index. Thus, the predefined ≤0.5-mm category represents a predefined severity group, whereas the Youden-derived cut-off represents the continuous measurement value that maximized the combined sensitivity and specificity for discrimination of the binary reference-standard classification.

For the primary binary diagnostic-accuracy analysis, classifications were based on the independently recorded binary interpretations of the three observers and combined using site-level majority vote; they were not generated by dichotomizing the mean continuous bone-loss measurement. The mean continuous measurement across the three observers was used only as the site-level predictor for the primary ROC analysis. Reader-specific ROC analyses used each observer's original continuous estimated marginal bone-loss measurement separately and did not alter the primary majority-vote diagnostic classifications.

### Clustered multi-reader analysis

Because the same implant sites were assessed using both imaging modalities and 30 of the 100 patients contributed two implant sites, the multi-reader analysis accounted for clustering at the patient level. A generalized estimating equation (GEE) logistic model with robust sandwich standard errors was used, with diagnostic correctness as the binary outcome. Imaging modality, observer identity, implant location, and reference-standard bone-loss severity were included as adjustment factors, with patient specified as the clustering variable. Observer experience was not included as an inferential predictor because each experience category was represented by only one observer and was therefore completely confounded with observer identity.

The patient-clustered model demonstrated significantly higher odds of correct diagnostic classification with CBCT than with digital periapical radiography (OR = 1.97, 95% CI: 1.16–3.34; *p* = 0.012). Observer-specific differences were presented descriptively and were not interpreted as independent effects of professional experience.

### Observer-specific diagnostic performance

To characterize reader-level variability, diagnostic performance was additionally evaluated separately for each of the three observers using their original binary interpretations of the CBCT and digital periapical radiographic examinations. The two implant sites with inconclusive reference-standard classifications were excluded from these analyses. Accordingly, each observer contributed 128 evaluable implant-site assessments for each imaging modality (Table 5).

**Table 5:** Observer-specific diagnostic performance of CBCT and digital periapical radiography.

Observer	Modality	Sensitivity, % (95% CI)	Specificity, % (95% CI)	PPV, % (95% CI)	NPV, % (95% CI)	Accuracy, % (95% CI)
Expert oral radiologist	CBCT	94.5 (86.7–97.8)	96.4 (87.7–99.0)	97.2 (90.3–99.2)	93.0 (83.3–97.2)	95.3 (90.2–97.8)
Expert oral radiologist	DPR	74.0 (62.9–82.7)	100.0 (93.5–100.0)	100.0 (93.4–100.0)	74.3 (63.3–82.9)	85.2 (78.0–90.3)
General dentist	CBCT	84.9 (75.0–91.4)	92.7 (82.7–97.1)	93.9 (85.4–97.6)	82.3 (71.0–89.8)	88.3 (81.6–92.8)
General dentist	DPR	76.7 (65.8–84.9)	98.2 (90.4–99.7)	98.2 (90.7–99.7)	76.1 (65.0–84.5)	85.9 (78.9–90.9)
Postgraduate trainee	CBCT	87.7 (78.2–93.4)	89.1 (78.2–94.9)	91.4 (82.5–96.0)	84.5 (73.1–91.6)	88.3 (81.6–92.8)
Postgraduate trainee	DPR	74.0 (62.9–82.7)	83.6 (71.7–91.1)	85.7 (75.0–92.3)	70.8 (58.8–80.4)	78.1 (70.2–84.4)

Values are based on the original binary interpretations of each observer. Two implant sites with inconclusive reference-standard classifications were excluded, leaving 128 evaluable sites. Confidence intervals were calculated using the Wilson method. Observer assessments were not treated as independent patient-level observations.

The expert oral radiologist demonstrated a sensitivity of 94.5% (69/73; 95% CI: 86.7%–97.8%), specificity of 96.4% (53/55; 95% CI: 87.7%–99.0%), positive predictive value (PPV) of 97.2% (69/71; 95% CI: 90.3%–99.2%), negative predictive value (NPV) of 93.0% (53/57; 95% CI: 83.3%–97.2%), and overall diagnostic accuracy of 95.3% (122/128; 95% CI: 90.2%–97.8%) with CBCT. With digital periapical radiography, the corresponding values were 74.0% sensitivity (54/73; 95% CI: 62.9%–82.7%), 100.0% specificity (55/55; 95% CI: 93.5%–100.0%), 100.0% PPV (54/54; 95% CI: 93.4%–100.0%), 74.3% NPV (55/74; 95% CI: 63.3%–82.9%), and 85.2% diagnostic accuracy (109/128; 95% CI: 78.0%–90.3%).

The general dentist demonstrated a sensitivity of 84.9% (62/73; 95% CI: 75.0%–91.4%), specificity of 92.7% (51/55; 95% CI: 82.7%–97.1%), PPV of 93.9% (62/66; 95% CI: 85.4%–97.6%), NPV of 82.3% (51/62; 95% CI: 71.0%–89.8%), and diagnostic accuracy of 88.3% (113/128; 95% CI: 81.6%–92.8%) with CBCT. With digital periapical radiography, sensitivity was 76.7% (56/73; 95% CI: 65.8%–84.9%), specificity was 98.2% (54/55; 95% CI: 90.4%–99.7%), PPV was 98.2% (56/57; 95% CI: 90.7%–99.7%), NPV was 76.1% (54/71; 95% CI: 65.0%–84.5%), and diagnostic accuracy was 85.9% (110/128; 95% CI: 78.9%–90.9%).

The postgraduate trainee demonstrated a sensitivity of 87.7% (64/73; 95% CI: 78.2%–93.4%), specificity of 89.1% (49/55; 95% CI: 78.2%–94.9%), PPV of 91.4% (64/70; 95% CI: 82.5%–96.0%), NPV of 84.5% (49/58; 95% CI: 73.1%–91.6%), and diagnostic accuracy of 88.3% (113/128; 95% CI: 81.6%–92.8%) with CBCT. With digital periapical radiography, sensitivity was 74.0% (54/73; 95% CI: 62.9%–82.7%), specificity was 83.6% (46/55; 95% CI: 71.7%–91.1%), PPV was 85.7% (54/63; 95% CI: 75.0%–92.3%), NPV was 70.8% (46/65; 95% CI: 58.8%–80.4%), and diagnostic accuracy was 78.1% (100/128; 95% CI: 70.2%–84.4%).

Across all three observers, CBCT demonstrated higher sensitivity and overall diagnostic accuracy than digital periapical radiography. The magnitude of the difference varied across observers, with the largest absolute differences in sensitivity and diagnostic accuracy observed for the expert oral radiologist. Digital periapical radiography generally showed higher specificity, particularly for the expert oral radiologist and general dentist. These observer-specific findings were consistent with the direction of the primary site-level majority-vote analysis and demonstrate reader-level variability in diagnostic performance (Table 5, Figure 3). Because each observer represented a different experience category, these differences cannot be interpreted as independent effects of professional experience.

**Figure 3:**
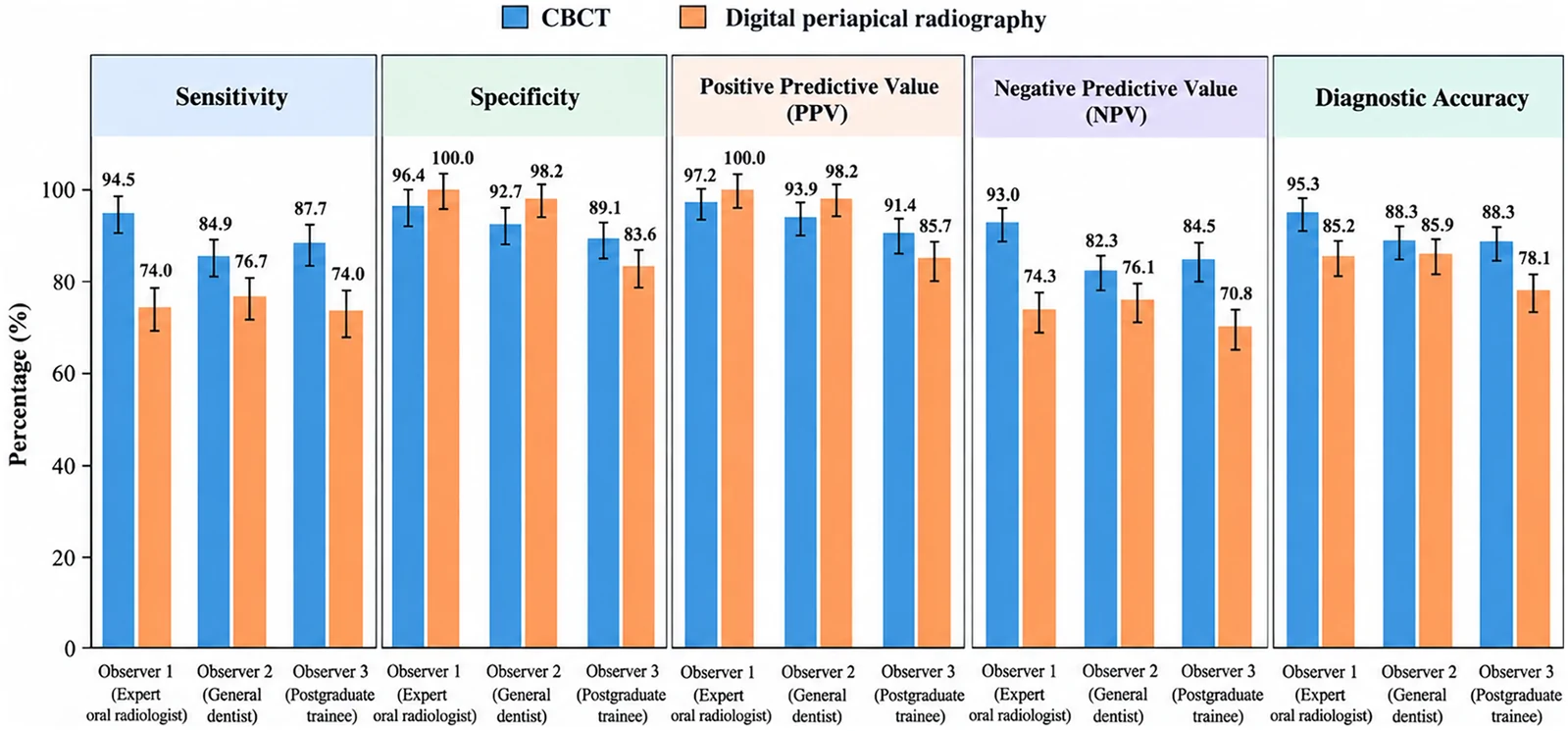
Observer-wise comparison of diagnostic performance between CBCT and digital periapical radiography. CBCT demonstrated higher sensitivity and overall diagnostic accuracy across the three observers, whereas digital periapical radiography generally demonstrated higher specificity. Observer-specific diagnostic performance is presented descriptively; differences among observers should not be interpreted as independent effects of professional experience because observer identity and experience category were completely confounded.

### Threshold-based analysis by bone-loss severity

Severity-specific analysis was performed using the predefined bone-loss categories of ≤0.5 mm, >0.5–1 mm, and >1 mm. These predefined categories were specified independently of the continuous ROC analysis and its Youden-index-derived diagnostic threshold. Representative CBCT and digital periapical radiographic images illustrating the predefined bone-loss severity categories are presented in Figure 4.

**Figure 4:**
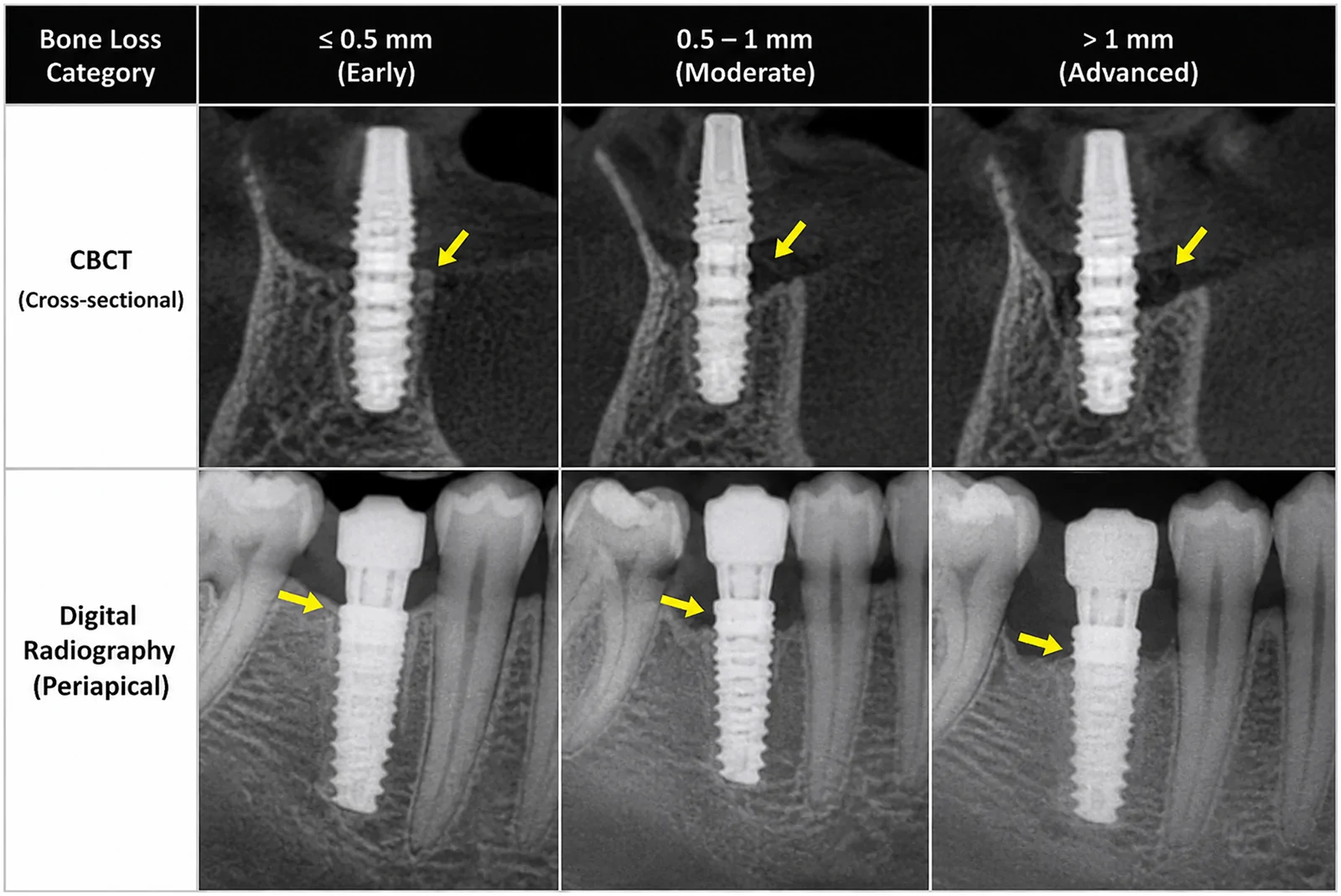
Representative CBCT and digital radiographic images demonstrating different peri-implant bone-loss severity categories (≤0.5 mm, >0.5–1 mm, and >1 mm).

Among the 15 reference-standard-positive implant sites with ≤0.5 mm of peri-implant marginal bone loss, CBCT demonstrated a sensitivity of 100.0% (15/15), compared with 46.7% (7/15) for digital periapical radiography (Table 6, Figure 5).

**Table 6:** Sensitivity of CBCT and digital periapical radiography according to predefined bone-loss severity.

Bone-loss category	CBCT sensitivity, % (95% CI)	Digital periapical radiography sensitivity, % (95% CI)
≤0.5 mm (*n* = 15)	100.0 (79.6–100.0)	46.7 (24.5–70.5)
>0.5–1 mm (*n* = 40)	95.0 (83.5–98.6)	80.0 (65.3–89.1)
>1 mm (*n* = 18)	100.0 (82.4–100.0)	100.0 (82.4–100.0)

The bone-loss categories were predefined severity groups based on the calculated change in marginal bone level and were not ROC-derived diagnostic thresholds. Sensitivity was calculated separately within each reference-standard-positive severity group. The denominators were 15 implant sites for ≤0.5 mm, 40 sites for >0.5–1 mm, and 18 sites for >1 mm, corresponding to all 73 reference-standard-positive implant sites. Ninety-five percent confidence intervals were calculated using the Wilson method. The continuous estimated marginal bone-loss measurement, rather than the categorical severity groups or 5-point confidence scores, was used as the predictor for ROC analysis. The optimal data-driven diagnostic cut-off was determined separately using the Youden index and should not be interpreted as equivalent to the predefined severity categories.

**Figure 5:**
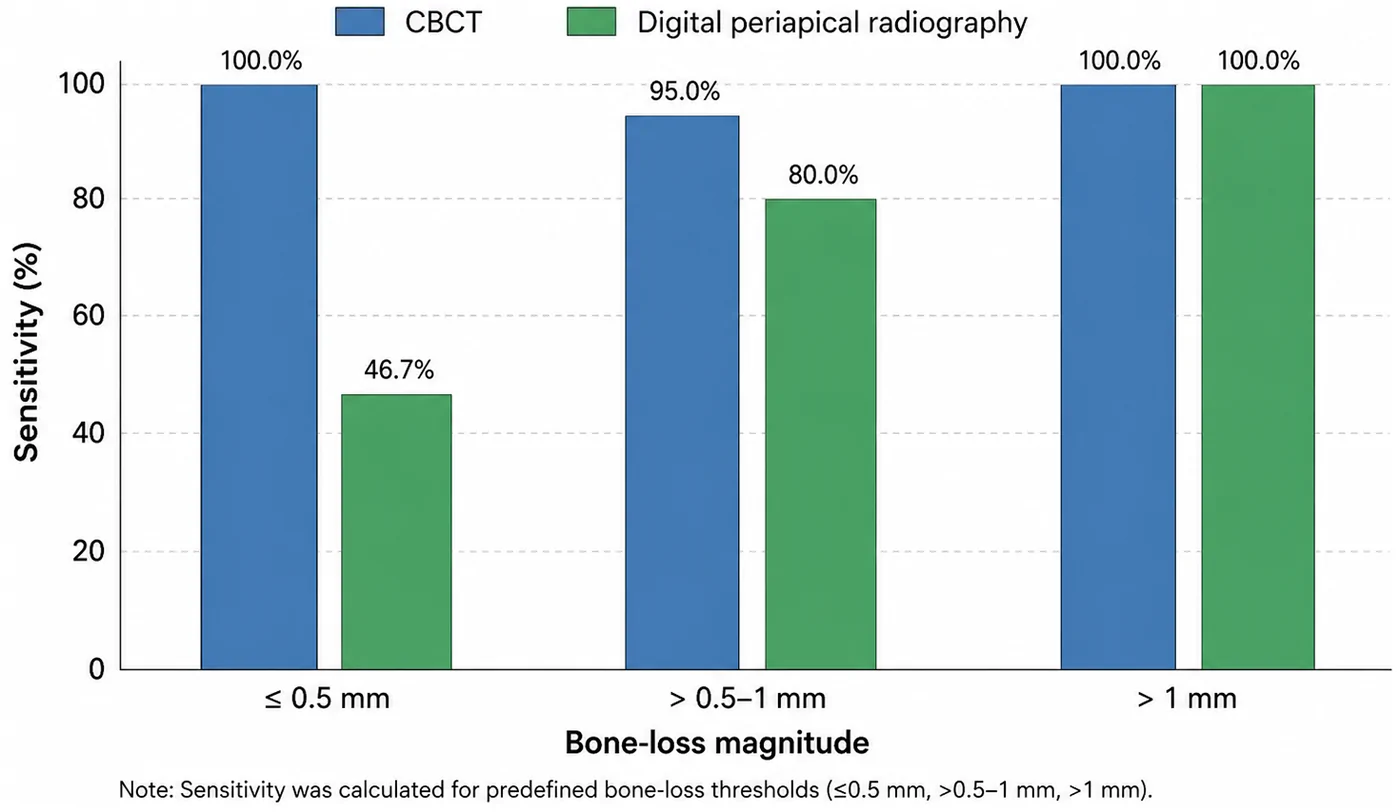
Sensitivity of CBCT and digital periapical radiography according to predefined peri-implant marginal bone-loss severity. Sensitivity was calculated within each reference-standard-positive severity category: ≤0.5 mm (*n* = 15), >0.5–1 mm (*n* = 40), and >1 mm (*n* = 18). CBCT demonstrated higher sensitivity than digital periapical radiography for ≤0.5 mm and >0.5–1 mm bone-loss categories, whereas both modalities demonstrated 100% sensitivity for defects >1 mm. The predefined severity categories were independent of the continuous ROC analysis and were not derived from the Youden-index threshold.

For peri-implant marginal bone loss of >0.5–1 mm, CBCT demonstrated a sensitivity of 95.0% (38/40), compared with 80.0% (32/40) for digital periapical radiography. For defects >1 mm, sensitivity was 100.0% (18/18) for both CBCT and digital periapical radiography (Table 6, Figure 5).

The largest observed difference between the modalities was in the ≤0.5 mm category, where CBCT sensitivity was 100.0% compared with 46.7% for digital periapical radiography. A smaller difference was observed in the **>**0.5–1 mm category, whereas both modalities demonstrated 100.0% sensitivity for defects >1 mm. These severity-specific estimates should be interpreted cautiously because the ≤0.5 mm category contained only 15 reference-standard-positive sites, and very small changes in marginal bone level may be affected by measurement variability and clinical/radiographic uncertainty. The findings therefore indicate a higher observed sensitivity of CBCT for the smallest predefined bone-loss category but should not be interpreted as establishing that CBCT can reliably detect biologically meaningful disease progression at or below 0.5 mm. The Youden-index-derived optimal diagnostic cut-off from the continuous ROC analysis was evaluated separately and should not be interpreted as equivalent to the predefined severity categories.

### Observer agreement and repeatability

Interobserver agreement was assessed separately for each pair of observers for CBCT and digital periapical radiography using linear-weighted Cohen's kappa for the four-level ordinal diagnostic classifications. The individual pairwise estimates were examined in addition to the mean pairwise kappa to characterize agreement between observer pairs.

For CBCT, the weighted kappa values were 0.83 for Observer 1 vs. Observer 2, 0.85 for Observer 1 vs. Observer 3, and 0.86 for Observer 2 vs. Observer 3, with a mean pairwise kappa of 0.85 (95% CI: 0.78–0.91). For digital periapical radiography, the corresponding values were 0.69, 0.68, and 0.64, respectively, with a mean pairwise kappa of 0.67 (95% CI: 0.58–0.75). Thus, interobserver agreement was consistently higher for CBCT than for digital periapical radiography.

Intraobserver repeatability was evaluated using the continuous estimated marginal bone-loss measurements obtained during the initial and 2-week repeat assessments. Observer-specific ICC(2,1) values for CBCT were 0.92 for the expert oral radiologist, 0.93 for the general dentist, and 0.92 for the postgraduate trainee. For digital periapical radiography, the corresponding ICC(2,1) values were 0.76, 0.79, and 0.77, respectively. These findings indicate high intraobserver repeatability for CBCT across all three observers and comparatively lower repeatability for digital periapical radiography. The overall modality-level ICC values were 0.93 (95% CI: 0.90–0.94) for CBCT and 0.77 (95% CI: 0.70–0.83) for digital periapical radiography (Table 7, Figure 6).

**Table 7:** Interobserver and intraobserver agreement analysis.

Agreement parameter	CBCT	Digital periapical radiography
Interobserver weighted Cohen's *κ*, mean of 3 pairwise comparisons (95% CI)	0.85 (0.78–0.91)	0.67 (0.58–0.75)
Intraobserver ICC(2,1), absolute agreement (95% CI)	0.93 (0.90–0.94)	0.77 (0.70–0.83)

Interobserver agreement was assessed using linear-weighted Cohen's kappa for the four-level ordinal bone-loss categories (no bone loss, ≤0.5 mm, >0.5–1 mm, and >1 mm). The reported kappa represents the mean of the three pairwise observer comparisons. Intraobserver reliability was assessed using a two-way random-effects, single-measure ICC for absolute agreement [ICC(2,1)] based on the continuous estimated marginal bone-loss measurements from the initial and repeat assessments. Ninety-five percent confidence intervals were obtained by bootstrap resampling at the implant-site level.

**Figure 6:**
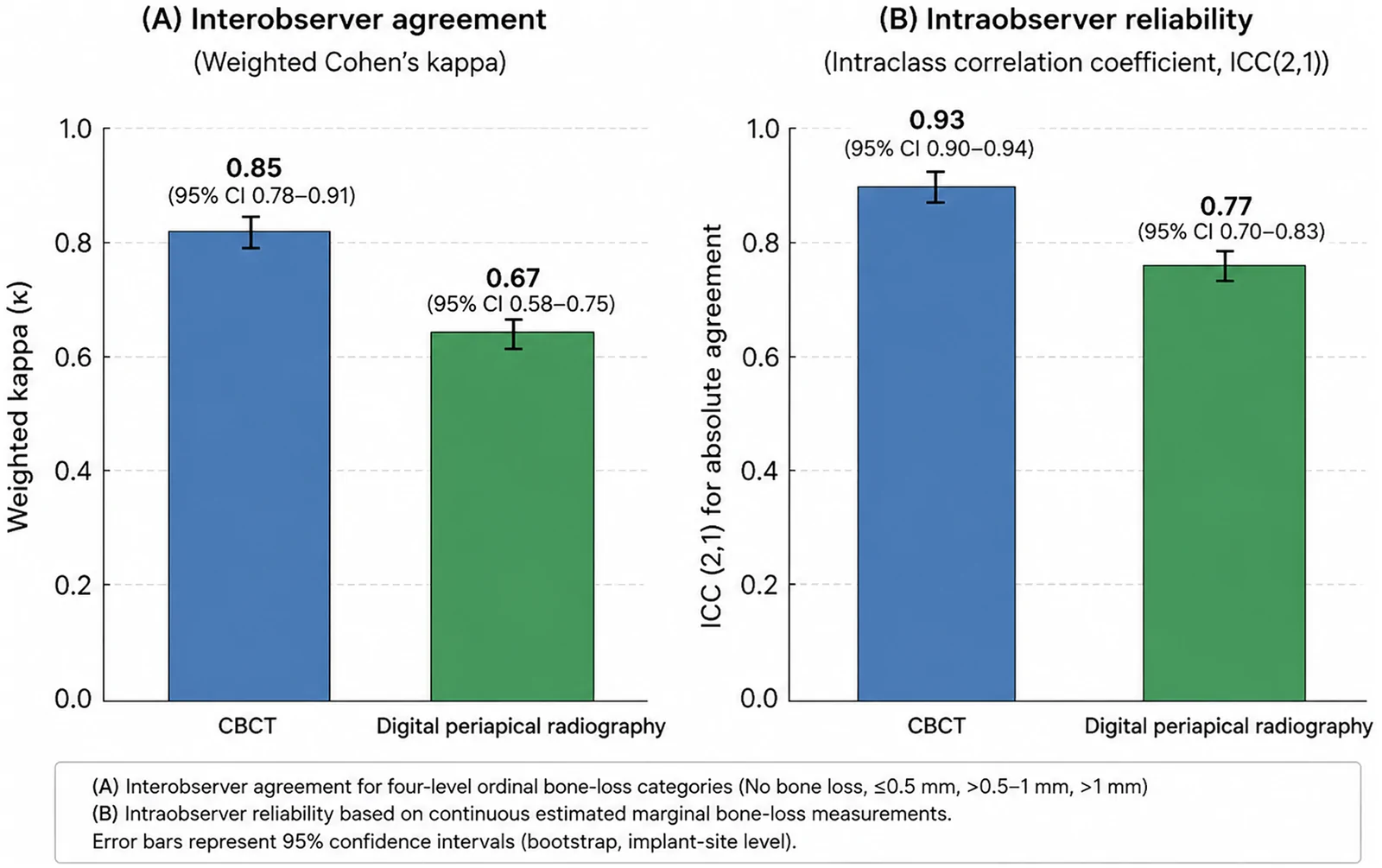
Agreement analysis comparing interobserver agreement and intraobserver reproducibility between CBCT and digital periapical radiography.

### Sensitivity and specificity analysis

Sensitivity and specificity were compared between CBCT and digital periapical radiography using paired implant-site classifications. Among the reference-standard-positive implant sites, CBCT demonstrated higher sensitivity than digital periapical radiography (97.3% vs. 78.1%). Among reference-standard-negative sites, digital periapical radiography demonstrated slightly higher specificity than CBCT (100.0% vs. 96.4%).

The paired binary classifications from the two imaging modalities were further examined descriptively. Among the 128 evaluable implant sites, the two modalities produced discordant classifications in 18 sites, comprising 17 sites classified as positive by CBCT but negative by digital periapical radiography and 1 site classified as negative by CBCT but positive by digital periapical radiography. Because some patients contributed more than one implant site, the inferential comparison of diagnostic correctness was performed using a patient-clustered generalized estimating equation (GEE) logistic model with robust standard errors, rather than a conventional McNemar test. In this model, diagnostic correctness was the binary outcome and imaging modality was the principal predictor, with patient specified as the clustering variable. CBCT demonstrated significantly higher odds of correct diagnostic classification than digital periapical radiography (OR = 1.97, 95% CI: 1.16–3.34; *p* = 0.012).

Severity-specific sensitivity results are presented separately in the severity-specific analysis above; therefore, the present section focuses on overall paired sensitivity, specificity, and the patient-clustered inferential comparison.

### Exploratory analysis according to imaging interval

An exploratory subgroup analysis was performed among implant sites with a shorter interval of ≤1 month between CBCT and digital periapical radiography. Among the 10 evaluable implant sites in this subgroup, 6 were classified as reference-standard positive and 4 as reference-standard negative. CBCT demonstrated a sensitivity of 100.0% (6/6; 95% CI: 61.0–100.0%), specificity of 100.0% (4/4; 95% CI: 51.0–100.0%), and diagnostic accuracy of 100.0% (10/10; 95% CI: 72.2–100.0%). Digital periapical radiography demonstrated a sensitivity of 100.0% (6/6; 95% CI: 61.0–100.0%), specificity of 100.0% (4/4; 95% CI: 51.0–100.0%), and diagnostic accuracy of 100.0% (10/10; 95% CI: 72.2–100.0%).

Although the observed estimates were identical for the two modalities in this small subgroup, the analysis was based on only 10 evaluable implant sites and therefore had limited statistical precision. The wide confidence intervals and small subgroup size preclude a reliable assessment of whether a shorter imaging interval influenced diagnostic performance or the relative performance of the two modalities. Accordingly, this exploratory analysis should be considered inconclusive and should not be interpreted as evidence that the imaging interval had no material effect.

### Sensitivity analysis restricted to surgically verified sites

A prespecified sensitivity analysis restricted to the 45 surgically verified implant sites was performed to assess the robustness of the diagnostic-accuracy estimates with respect to reference-standard composition. One surgically verified site had an inconclusive reference-standard classification and was therefore excluded from analyses requiring a definitive binary reference outcome, leaving 44 evaluable sites. In this subgroup, CBCT demonstrated a sensitivity of 96.6% (28/29; 95% CI: 82.8%–99.4%), specificity of 100.0% (15/15; 95% CI: 79.6%–100.0%), and overall diagnostic accuracy of 97.7% (43/44; 95% CI: 88.2%–99.6%). Digital periapical radiography demonstrated a sensitivity of 86.2% (25/29; 95% CI: 68.3%–94.8%), specificity of 100.0% (15/15; 95% CI: 79.6%–100.0%), and overall diagnostic accuracy of 90.9% (40/44; 95% CI: 79.1%–96.5%).

The direction of the findings was consistent with the primary analysis, with CBCT demonstrating higher sensitivity and overall diagnostic accuracy than digital periapical radiography, while both modalities demonstrated 100.0% specificity in the surgically verified subgroup. Given the relatively small number of surgically verified sites, these subgroup estimates should be interpreted as supportive rather than definitive.

### Observer confidence and imaging assessment

Observer-level assessment characteristics varied across the three observers. The expert oral radiologist demonstrated the highest diagnostic confidence across both imaging modalities, while CBCT assessments showed greater interobserver consistency than digital periapical radiography. Because each observer represented a different clinical-experience category, these observer-level differences cannot be interpreted as independent effects of professional experience.

Interobserver agreement for the ordinal bone-loss categories was high for CBCT, with a mean linear-weighted Cohen's kappa of 0.85 (95% CI: 0.78–0.91), compared with 0.67 (95% CI: 0.58–0.75) for digital periapical radiography. Repeatability of the continuous estimated marginal bone-loss measurements was high for CBCT, with ICC(2,1) = 0.93 (95% CI: 0.90–0.94), compared with ICC(2,1) = 0.77 (95% CI: 0.70–0.83) for digital periapical radiography (Table 7, Figure 7).

**Figure 7:**
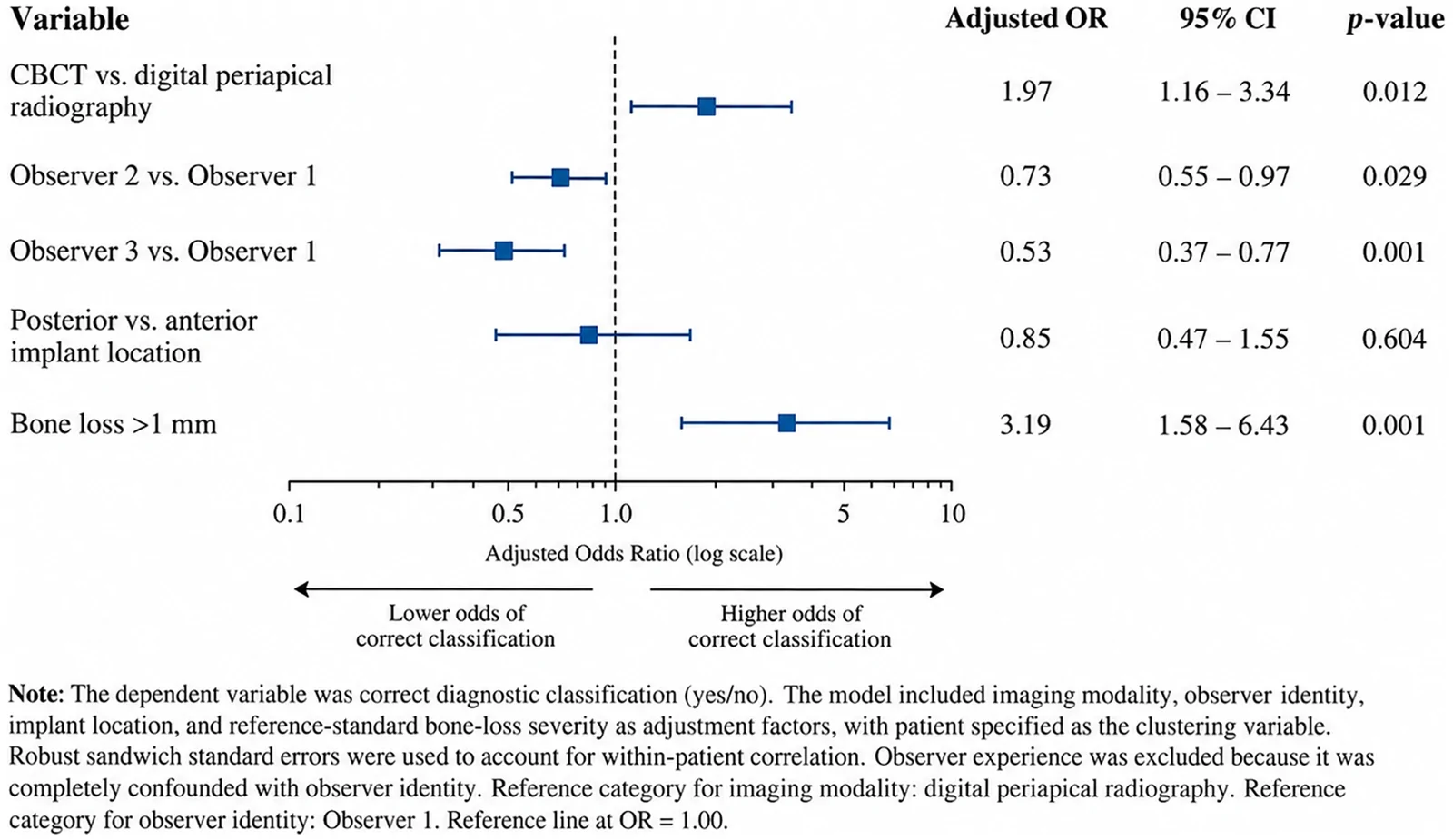
Forest plot of the patient-clustered multi-reader generalized estimating equation (GEE) logistic model for diagnostic correctness. The model included imaging modality, observer identity, implant location, and reference-standard bone-loss severity as adjustment factors and used patient as the clustering variable. Robust sandwich standard errors were used. Observer experience was excluded because it was completely confounded with observer identity.

Observer confidence scores were consistently higher for CBCT across all three observers (Table 8, Figure 8). The expert oral radiologist had the highest confidence scores for both CBCT (4.8 ± 0.3) and digital periapical radiography (4.1 ± 0.5), followed by the general dentist and postgraduate trainee. These differences were descriptive and were not interpreted as independent effects of professional experience.

**Table 8:** Observer confidence scores according to imaging modality.

Observer group	CBCT mean score	Digital periapical radiography mean score
Expert oral radiologist	4.8 ± 0.3	4.1 ± 0.5
General dentist	4.4 ± 0.5	3.7 ± 0.6
Postgraduate trainee	4.1 ± 0.6	3.3 ± 0.7

Confidence was recorded using a 5-point Likert scale, with higher scores indicating greater diagnostic confidence. Values are presented as mean ± standard deviation (SD).

**Figure 8:**
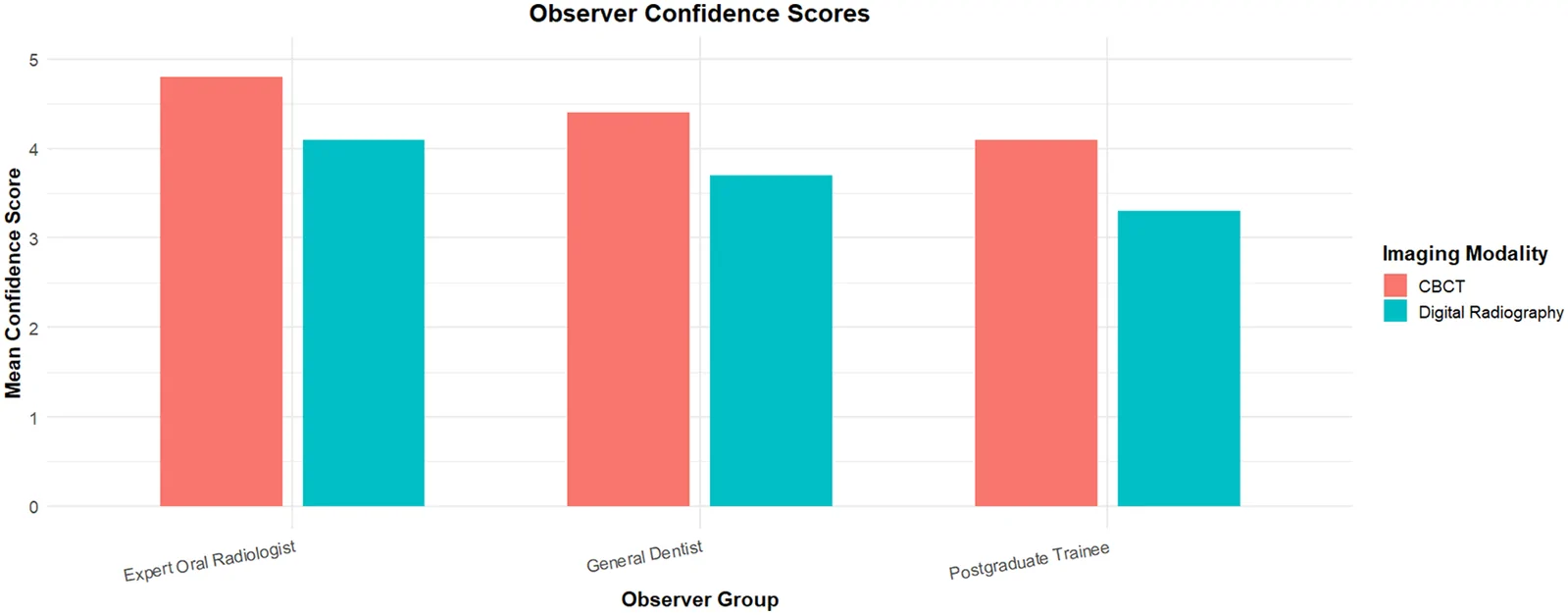
Mean observer confidence scores for CBCT and digital periapical radiography across the three observers. Confidence scores were higher for CBCT across all observers; differences among observers are presented descriptively and should not be interpreted as independent effects of professional experience.

### Patient-clustered multi-reader GEE analysis

A patient-clustered generalized estimating equation (GEE) logistic model was used to account for the multi-reader, multi-case structure and clustering arising from patients who contributed more than one implant site. Diagnostic correctness was specified as the binary outcome, with imaging modality as the principal predictor. Observer identity, implant location, and reference-standard bone-loss severity were included as adjustment factors, and patient was specified as the clustering variable. Observer experience was not included as an inferential predictor because each experience category was represented by only one observer and was therefore completely confounded with observer identity.

In the simpler patient-clustered GEE model containing imaging modality as the sole predictor, digital periapical radiography was associated with lower odds of correct classification than CBCT (OR, 0.51; 95% CI, 0.30–0.86; *p* = 0.012), corresponding to approximately 1.95-fold higher odds of correct classification with CBCT. After adjustment for observer identity, implant location, and reference-standard bone-loss severity, the modality effect remained essentially unchanged (digital periapical radiography vs. CBCT: OR, 0.51; 95% CI, 0.30–0.86; *p* = 0.012; equivalently, CBCT vs. digital periapical radiography: OR, 1.97; 95% CI, 1.16–3.34; *p* = 0.012). This consistency between the simpler and adjusted specifications supports the robustness of the observed modality effect.

The patient-clustered multi-reader GEE model demonstrated significantly higher odds of correct diagnostic classification with CBCT than with digital periapical radiography (adjusted OR, 1.97; 95% CI, 1.16–3.34; *p* = 0.012) (Table 9, Figure 7). Observer identity showed variation in diagnostic correctness, with lower odds for Observer 2 (OR, 0.73; 95% CI, 0.55–0.97; *p* = 0.029) and Observer 3 (OR, 0.53; 95% CI, 0.37–0.77; *p* = 0.001) compared with Observer 1. These differences were interpreted descriptively and not as independent effects of professional experience. Implant location was not significantly associated with diagnostic correctness (OR, 0.85; 95% CI, 0.47–1.55; *p* = 0.604), whereas bone-loss severity >1 mm was associated with higher odds of correct classification (OR, 3.19; 95% CI, 1.58–6.43; *p* = 0.001).

**Table 9:** Patient-clustered multi-reader generalized estimating equation (GEE) logistic analysis of diagnostic correctness.

Variable	Adjusted OR	95% CI	*p*-value
CBCT vs. digital periapical radiography	1.97	1.16–3.34	0.012
Observer 2 vs. Observer 1	0.73	0.55–0.97	0.029
Observer 3 vs. Observer 1	0.53	0.37–0.77	0.001
Posterior vs. anterior implant location	0.85	0.47–1.55	0.604
Bone loss >1 mm	3.19	1.58–6.43	0.001

The dependent variable was correct diagnostic classification (yes/no). The model included imaging modality, observer identity, implant location, and reference-standard bone-loss severity as adjustment factors, with patient specified as the clustering variable. Robust sandwich standard errors were used to account for within-patient correlation. The reference category for imaging modality was digital periapical radiography, and Observer 1 was the reference category for observer identity. Observer experience was not included as an inferential predictor because each experience category was represented by only one observer and was therefore completely confounded with observer identity. The analysis included 128 evaluable implant sites contributed by 100 unique patients, generating 768 observer-level modality assessments. Confidence intervals were derived from the robust sandwich covariance matrix clustered at the patient level.

Model convergence was achieved under the revised specification. The model used robust sandwich standard errors with patient as the clustering variable. Because the revised analysis did not include an observer random effect, no observer-level random-effect variance was estimated. Confidence intervals were derived from the robust sandwich covariance matrix clustered at the patient level, accounting for the 100 independent patient clusters rather than treating the 768 observer-level records as independent observations.

### Handling of indeterminate findings and missing data

All three observers provided definitive binary interpretations (positive or negative) for both CBCT and digital periapical radiography at each of the 128 implant sites with a definitive reference-standard classification. Consequently, no indeterminate index-test interpretations were present at the observer level, and no site lacked two concordant definitive observer interpretations. The site-level binary classification was therefore determined by the prespecified majority-vote rule (≥2 of 3 observers).

As a sensitivity analysis, sites with any indeterminate index-test interpretation were excluded; because no such sites were present, all 128 evaluable sites were retained and the results were identical to those of the primary analysis. A second sensitivity analysis treating indeterminate index-test interpretations as incorrect classifications was also performed; because no indeterminate index-test observations were present, no observations required reclassification and the results were identical to the primary analysis.

The two sites with inconclusive reference-standard classifications were excluded from analyses requiring a definitive binary reference outcome. Missing observations were handled using complete-case analysis for the relevant analysis.

### Adverse events

No adverse events related to CBCT acquisition, digital periapical radiography, or the reference-standard assessment were identified in the available retrospective study records.

## Discussion

The present retrospective diagnostic accuracy study demonstrated that CBCT provided higher diagnostic performance than digital periapical radiography under the conditions of this study for the detection of peri-implant marginal bone loss. CBCT showed higher sensitivity and overall diagnostic accuracy, greater discriminatory performance on ROC analysis, higher interobserver agreement, and greater intraobserver repeatability, while observer confidence scores were also higher for CBCT. These findings were particularly evident for small amounts of radiographic bone loss, with CBCT demonstrating higher observed sensitivity than digital periapical radiography for the predefined ≤0.5 mm and >0.5–1 mm categories. However, these findings should be interpreted as evidence of improved radiographic detectability under the evaluated imaging conditions rather than proof that CBCT identifies biologically progressive disease at the sub-millimeter scale. The ≤0.5 mm subgroup contained only 15 reference-standard-positive sites, and small changes in marginal bone level may be affected by measurement variability and clinical uncertainty. These findings support the established role of three-dimensional imaging as an adjunct to conventional intraoral radiography in selected peri-implant diagnostic situations (13–15).

Peri-implant diseases remain among the most important biological complications affecting long-term implant success and survival. Early identification of peri-implant bone changes is clinically relevant because disease progression may occur before obvious clinical manifestations become evident. Progressive marginal bone loss is an important feature of peri-implant disease and may be associated with peri-implantitis and, in advanced cases, implant failure (16, 17). Conventional digital radiography remains the most commonly used imaging modality for routine implant follow-up because of its accessibility, low radiation dose, rapid acquisition, and cost-effectiveness. However, its inherent two-dimensional limitations reduce its ability to accurately visualize buccal and lingual osseous defects because of anatomical superimposition and the restricted three-dimensional information provided by projection imaging (18, 19).

In the present study, CBCT demonstrated a sensitivity of 97.3% compared with 78.1% for digital periapical radiography and an overall diagnostic accuracy of 96.9% compared with 87.5%, respectively. The primary site-level ROC analysis also demonstrated greater discriminatory performance with CBCT than with digital periapical radiography (AUC, 0.91 vs. 0.78; DeLong test, *p* < 0.001). The patient-clustered multi-reader GEE analysis further supported the diagnostic advantage of CBCT, with significantly higher odds of correct classification compared with digital periapical radiography (adjusted OR = 1.97, 95% CI: 1.16–3.34; *p* = 0.012). Importantly, this inferential analysis accounted for the clustering introduced by patients who contributed more than one implant site and incorporated observer identity without treating observer experience as an independent predictor.

In the present study, digital periapical radiography demonstrated substantially lower sensitivity for detecting reference-standard-positive sites with ≤0.5 mm of marginal bone loss compared with CBCT. These findings are consistent with previous investigations demonstrating limitations of intraoral radiography in the detection of peri-implant osseous defects, particularly when defect morphology, location, and other imaging conditions are unfavorable (20, 21). Dave et al. reported that CBCT showed significantly improved detection of peri-implant defects compared with conventional periapical radiography, particularly in early osseous alterations (5). Similarly, Song et al. demonstrated superior diagnostic accuracy of CBCT compared with intraoral imaging for peri-implant bone defect assessment, especially in clinically challenging defect configurations (6). More recent systematic reviews and meta-analyses have also supported the diagnostic utility of CBCT for peri-implant bone-defect assessment, while emphasizing heterogeneity according to defect type, implant material, imaging protocol, and anatomical location (22, 23).

An important consideration is the heterogeneous composition of the reference standard. Surgical verification was available for 45 implant sites, of which 44 had a definitive binary reference classification. In this surgically verified subgroup, CBCT maintained higher sensitivity (96.6% vs. 86.2%) and overall diagnostic accuracy (97.7% vs. 90.9%) than digital periapical radiography, while both modalities demonstrated 100.0% specificity. These findings provide supportive evidence that the primary results were not solely driven by the clinical/longitudinal composite reference standard. Nevertheless, the majority of sites were classified using the composite approach, and potential incorporation of digital periapical radiographic information into its longitudinal component could not be excluded retrospectively. Differential-verification and residual incorporation bias therefore remain possible, and the primary diagnostic estimates should be interpreted accordingly.

The particularly high sensitivity observed with CBCT for the predefined ≤0.5 mm severity category requires cautious interpretation. A voxel size of 0.20 mm represents the sampling dimension of the reconstructed image and should not be equated with true spatial resolution or with the ability to measure a biological change of the same magnitude. Around titanium implants, beam-hardening, scatter, partial-volume effects, and other reconstruction-related factors can affect the apparent position of the crestal bone. Therefore, identification of a radiographic difference of a few tenths of a millimeter should be interpreted as improved radiographic detectability under the imaging conditions used in this study, rather than as definitive evidence of biologically meaningful bone loss or disease progression. This distinction is particularly important when interpreting sub-millimeter findings and underscores the need for prospective longitudinal validation.

The exploratory analysis restricted to an imaging interval of ≤1 month was based on only 10 evaluable sites and therefore had limited statistical precision. Although both modalities achieved 100% observed sensitivity, specificity, and diagnostic accuracy in this subgroup, the wide confidence intervals and small sample size preclude a reliable conclusion regarding whether temporal separation between examinations influenced diagnostic performance or the relative performance of the two modalities. Accordingly, these findings should be regarded as inconclusive rather than as evidence that imaging interval had no material effect.

Receiver operating characteristic analysis further supported the superior discriminatory performance of CBCT, with an AUC of 0.91 compared with 0.78 for digital periapical radiography. These findings are consistent with previous studies demonstrating the diagnostic advantages of CBCT for selected peri-implant defect configurations, including dehiscence, fenestration, and circumferential defects (24, 25). De-Azevedo-Vaz et al. demonstrated that CBCT accuracy is influenced by defect size and localization, supporting the severity-specific findings observed in the present study (16). Similarly, Kühl et al. reported differences in the detectability of peri-implant bone defects among radiographic techniques (26). The present study additionally combined ROC analysis, predefined severity-specific assessment, observer variability evaluation, and patient-clustered GEE analysis to examine diagnostic performance across several complementary dimensions.

One of the principal strengths of the present investigation was the severity-specific analysis of defect detectability. CBCT demonstrated greater sensitivity than digital periapical radiography for lesions in the ≤0.5 mm and >0.5–1 mm predefined severity categories, suggesting that three-dimensional imaging may facilitate the radiographic identification of smaller marginal bone changes. However, the predefined severity categories represent radiographic measurement groups and should not be interpreted as biological thresholds for disease onset or progression. In particular, the ≤0.5 mm findings should be considered evidence of differences in image-based detectability rather than evidence that CBCT can establish clinically meaningful disease progression at 0.5 mm. Previous studies evaluating peri-implant imaging have frequently focused on advanced defects or artificially created lesions, thereby limiting direct clinical translation (22, 23, 25). The present study adds clinical retrospective evidence on the comparative detection of marginal bone changes across predefined severity categories, while prospective longitudinal studies remain necessary to establish their biological and prognostic significance.

Observer variability analysis further demonstrated higher agreement and reproducibility for CBCT than for digital periapical radiography. In addition, observer confidence scores were higher for CBCT across all three observers. These findings suggest that CBCT interpretation may facilitate more consistent image-based assessment under the standardized viewing conditions used in this study. This is broadly compatible with previous evidence showing that CBCT performance may be less affected than periapical radiography by examiner experience and by certain defect-related imaging variables (27, 28). Nevertheless, higher observer confidence should not itself be interpreted as evidence of greater biological diagnostic validity, particularly when interpreting sub-millimeter findings. The reader-specific analyses also showed higher CBCT AUCs than digital periapical radiography for each of the three observers, indicating that the overall discriminatory advantage of CBCT was not attributable to a single reader. Because each experience level was represented by one observer, however, observer experience remains completely confounded with observer identity. Accordingly, differences between observers should be interpreted descriptively rather than as independent effects of professional experience.

The patient-clustered multi-reader GEE analysis demonstrated that CBCT was associated with significantly higher odds of correct peri-implant bone-loss classification than digital periapical radiography (adjusted OR = 1.97, 95% CI: 1.16–3.34; *p* = 0.012), after accounting for observer identity, implant location, and reference-standard bone-loss severity. This analysis accounted for the clustering of implant sites within patients and the repeated assessments generated by the multi-reader design. The findings reinforce the overall diagnostic advantage of CBCT while avoiding an inappropriate attribution of differences to professional experience, which was completely confounded with observer identity in this three-observer study. The diagnostic advantage of CBCT was particularly evident for the smaller predefined bone-loss categories, although these findings should be interpreted cautiously because very small radiographic changes may be affected by measurement uncertainty and do not necessarily represent biologically progressive disease. Accordingly, the findings are most appropriately interpreted as supporting the potential utility of CBCT for selected diagnostic situations in which conventional radiographic assessment is limited (29). Importantly, the GEE estimate represents an adjusted association rather than a causal effect.

Despite these advantages, routine use of CBCT for all implant follow-up examinations cannot currently be justified. CBCT involves ionizing radiation and may require greater resources than conventional radiography, while implant-related beam-hardening artifacts can affect image interpretation (8, 9, 17, 20). Metallic artifacts surrounding titanium implants may impair visualization and reduce diagnostic precision in certain clinical situations. These considerations are particularly relevant when imaging is performed for routine surveillance rather than to address a specific unresolved diagnostic question. Contemporary radiation-protection principles, including as low as reasonably achievable (ALARA) and as low as diagnostically acceptable (ALADA), emphasize careful justification and optimization of CBCT acquisition. Therefore, CBCT should be used selectively when clinically justified, particularly when conventional radiographic findings are equivocal, when early peri-implant disease is clinically suspected, or when detailed buccolingual assessment of a suspected defect is required. The broader clinical management of implant-related complications should also take into account clinical findings and prosthetic factors rather than relying on imaging alone (30, 31).

### Limitations

Several limitations of the present study should be acknowledged. First, the retrospective design may have introduced selection bias and limited standardization of imaging acquisition parameters.

Second, although the reference standard was designed to minimize incorporation bias, a uniform independent reference standard was not available for all implant sites because surgical verification was available for only 45 of the 130 sites. For the remaining 85 sites, the clinical/longitudinal composite reference standard was established without incorporating the CBCT diagnostic interpretation, thereby reducing the potential for direct incorporation of the CBCT index test into the reference classification. However, the retrospective records did not permit complete confirmation that digital periapical radiographic findings were absent from the longitudinal component of the composite reference standard. Consequently, residual incorporation and differential-verification bias cannot be completely excluded. Such bias may have influenced the diagnostic accuracy estimates. To assess the robustness of the findings with respect to reference-standard composition, a sensitivity analysis restricted to the surgically verified subgroup was performed. Among the 44 evaluable surgically verified sites, CBCT demonstrated a sensitivity of 96.6%, specificity of 100.0%, and diagnostic accuracy of 97.7%, compared with 86.2%, 100.0%, and 90.9%, respectively, for digital periapical radiography. The direction of the findings remained consistent with the primary analysis, although the relatively small size of the surgically verified subgroup limits the precision of these estimates.

Third, although the interval between CBCT and digital periapical radiography was restricted to ≤3 months, the two index examinations were not necessarily performed on the same day. Consequently, a small amount of true biological change in peri-implant marginal bone level could have occurred between the two examinations and may have contributed to some discordant imaging findings, particularly for very small changes in marginal bone level. An exploratory subgroup analysis restricted to implant sites with a shorter interval (≤1 month) included only 10 evaluable sites. Although both modalities showed 100% observed sensitivity, specificity, and diagnostic accuracy in this subgroup, the wide confidence intervals and small sample size preclude a reliable assessment of whether temporal separation influenced diagnostic performance or the relative performance of the two modalities. This analysis should therefore be considered inconclusive rather than evidence that imaging interval had no material effect.

Fourth, metal-related artifacts may have influenced CBCT interpretation despite the exclusion of cases with severe artifacts. Fifth, the study was conducted in a single institutional setting, which may limit the external generalizability of the findings. Sixth, only two of the 130 implant sites had inconclusive reference-standard classifications; these sites were excluded from analyses requiring a definitive binary reference outcome, leaving 128 evaluable sites. Although their exclusion is unlikely to materially alter the primary estimates, the small number of inconclusive cases precluded meaningful analysis of indeterminate reference-standard outcomes. In addition, the relatively small number of sites within individual severity categories, particularly the ≤0.5 mm (*n* = 15) and >1 mm (*n* = 18) groups, limits the precision with which severity-specific estimates can be generalized. The particularly high sensitivity observed for CBCT in the ≤0.5 mm category should therefore be interpreted cautiously and as evidence of radiographic detectability rather than proof of biologically meaningful disease progression.

Finally, the use of multiple observers and the contribution of multiple implant sites by some patients introduced a hierarchical data structure. This structure was addressed analytically using patient-clustered GEE with robust sandwich standard errors, with patient specified as the clustering variable. Nevertheless, only 100 unique patients contributed the 128 evaluable sites used in the binary diagnostic analyses, and the multi-reader structure involved only three observers. Observer experience was completely confounded with observer identity because each experience category was represented by one observer; consequently, experience could not be evaluated as an independent inferential predictor. Observer-specific differences were therefore presented descriptively rather than interpreted as independent effects of professional experience.

A major strength of the present study was the use of a real-world retrospective clinical dataset combined with blinded multi-observer assessment, paired evaluation of both imaging modalities, continuous-measurement ROC analysis, severity-specific assessment, observer agreement and repeatability analysis, and patient-clustered GEE modeling. The inclusion of both patient- and implant-site-level information and the use of blinded multi-observer assessment further strengthened the evaluation of diagnostic performance under clinical imaging conditions. The use of majority-vote binary classifications for the primary diagnostic analysis while retaining continuous bone-loss measurements for ROC analysis also allowed complementary assessment of categorical diagnostic performance and continuous discriminatory ability.

Future prospective multicenter investigations using standardized imaging protocols and longitudinal peri-implant monitoring are warranted to further validate the findings of the present study. Such studies should distinguish radiographic detectability from true longitudinal biological bone loss and should incorporate standardized reference measurements where feasible. In addition, advances in low-dose CBCT protocols, image reconstruction algorithms, and artificial intelligence-assisted interpretation may further optimize peri-implant imaging strategies in clinical practice. The findings of the present study support selective rather than routine consideration of CBCT for peri-implant monitoring, particularly in patients with suspected early peri-implant disease or equivocal conventional radiographic findings. Future studies should also use temporally matched examinations, preferably obtained during the same clinical assessment, and should include multiple observers within each experience category to allow observer experience to be evaluated separately from observer-specific effects.

## Conclusion

Under the conditions of this retrospective study, CBCT demonstrated higher diagnostic performance than digital periapical radiography, with a consistent direction of effect in the sensitivity analysis restricted to surgically verified sites. The advantage was particularly evident for the detection of smaller radiographic marginal bone changes. However, sub-millimeter findings should be interpreted as differences in radiographic detectability rather than definitive evidence of biologically meaningful bone loss or disease progression, because voxel size does not represent true spatial resolution and implant-related imaging artifacts may affect measurements at this scale.

These findings support the selective use of CBCT when conventional periapical radiography is equivocal or when three-dimensional assessment of peri-implant structures is clinically indicated. They do not support routine CBCT screening of asymptomatic implant sites or routine CBCT use for all implant follow-up examinations, nor do they establish a definitive biological threshold for early peri-implant disease. CBCT should therefore be considered only when clinically justified, with appropriate radiation-protection, justification, and optimization principles applied. Prospective longitudinal studies using robust reference standards and temporally matched examinations are required to determine the clinical and prognostic significance of sub-millimeter radiographic changes.

## Data Availability

The original contributions presented in the study are included in the article/Supplementary Material, further inquiries can be directed to the corresponding author.
